# A semi-supervised Bayesian approach for simultaneous protein sub-cellular localisation assignment and novelty detection

**DOI:** 10.1371/journal.pcbi.1008288

**Published:** 2020-11-09

**Authors:** Oliver M. Crook, Aikaterini Geladaki, Daniel J. H. Nightingale, Owen L. Vennard, Kathryn S. Lilley, Laurent Gatto, Paul D. W. Kirk

**Affiliations:** 1 Cambridge Centre for Proteomics, Department of Biochemistry, University of Cambridge, Cambridge, UK; 2 MRC Biostatistics Unit, School of Clinical Medicine, University of Cambridge, Cambridge, UK; 3 Department of Genetics, Universtiy of Cambridge, Cambridge, UK; 4 de Duve Institute, UCLouvain, Avenue Hippocrate 75, 1200 Brussels, Belgium; 5 Cambridge Institute of Therapeutic Immunology & Infectious Disease (CITIID), Jeffrey Cheah Biomedical Centre, Cambridge Biomedical Campus, University of Cambridge, UK; 6 Milner Therapeutics Institute, Jeffrey Cheah Biomedical Centre, University of Cambridge, Puddicombe Way, Cambridge CB2 0AW, Cambridge, UK; University of Bologna, ITALY

## Abstract

The cell is compartmentalised into complex micro-environments allowing an array of specialised biological processes to be carried out in synchrony. Determining a protein’s sub-cellular localisation to one or more of these compartments can therefore be a first step in determining its function. High-throughput and high-accuracy mass spectrometry-based sub-cellular proteomic methods can now shed light on the localisation of thousands of proteins at once. Machine learning algorithms are then typically employed to make protein-organelle assignments. However, these algorithms are limited by insufficient and incomplete annotation. We propose a semi-supervised Bayesian approach to novelty detection, allowing the discovery of additional, previously unannotated sub-cellular niches. Inference in our model is performed in a Bayesian framework, allowing us to quantify uncertainty in the allocation of proteins to new sub-cellular niches, as well as in the number of newly discovered compartments. We apply our approach across 10 mass spectrometry based spatial proteomic datasets, representing a diverse range of experimental protocols. Application of our approach to *hyper*LOPIT datasets validates its utility by recovering enrichment with chromatin-associated proteins without annotation and uncovers sub-nuclear compartmentalisation which was not identified in the original analysis. Moreover, using sub-cellular proteomics data from *Saccharomyces cerevisiae*, we uncover a novel group of proteins trafficking from the ER to the early Golgi apparatus. Overall, we demonstrate the potential for novelty detection to yield biologically relevant niches that are missed by current approaches.

This is a *PLOS Computational Biology* Methods paper.

## Introduction

Aberrant protein sub-cellular localisation has been implicated in numerous diseases, including cancers [1], obesity [2], and multiple others [3]. Furthermore, recent estimates suggest that up to 50% of proteins reside in multiple locations with potentially different functions in each sub-cellular niche [4, 5]. Characterising the sub-cellular localisation of proteins is therefore of critical importance in order to understand the pathobiological mechanisms and aetiology of many diseases. Proteins are compartmentalised into sub-cellular niches, including organelles, sub-cellular structures, liquid phase droplets and protein complexes. These compartments ensure that the biochemical conditions for proteins to function correctly are met, and that they are in the proximity of interaction partners [6]. A common approach to map the global sub-cellular localisation of proteins is to couple gentle cell lysis with high-accuracy mass spectrometry (MS) [4, 7–9]. These methods are designed to yield fractions differentially enriched in the sub-cellular compartments rather than purifying the compartments into individual fractions. As such, these spatial proteomics approaches aim to interrogate the greatest number of sub-cellular niches possible by relying upon rigorous data analysis and interpretation [10, 11].

Current computational approaches in MS-based spatial proteomics utilise machine learning algorithms to make protein-organelle assignments (see [11] for an overview). Within this framework, novelty detection, the process of identifying differences between testing and training data, has multiple benefits. For model organisms with well annotated proteomes, novelty detection can potentially uncover groups of proteins with shared sub-cellular niches not described by the training data. Novelty detection can also prove useful in validating experimental design, either by demonstrating that contaminants have been removed or that increased resolution of organelle classes has been achieved by the experimental approach. For most non-model organisms, we have little *a priori* knowledge of their sub-cellular proteome organisation, making it challenging to curate the marker set (training dataset) from the literature [12]. In these cases, novelty detection can assist in annotating the spatial proteome. Crucially, if a dataset is insufficiently annotated, i.e sub-cellular niches detectable in the experimental data are missing from the marker set, then this leads to the classifier making erroneous assignments, resulting in inflated *false discovery rate* (FDR) and uncertainty estimates (where available). Thus, novelty detection is a useful feature for any classifier, even if novel niche detection is not a primary aim.

Previous efforts to discover novel niches within existing sub-cellular proteomics datasets have proved valuable. [13] presented a phenotype discovery algorithm called *phenoDisco* to detect novel sub-cellular niches and alleviate the issue of undiscovered phenotypes. The algorithm uses an iterative procedure and the *Bayesian Information Criterion* (BIC) [14] is employed to determine the number of newly detected phenotypes. Afterwards, the dataset can be re-annotated and a classifier employed to assign proteins to organelles, including those that have been newly detected. [13] applied their method on several datasets and discovered new organelle classes in *Arabidopsis* [15] and *Drosophila* [16]. This approach later successfully identified the trans-Golgi network (TGN) in *Arabidopsis* roots [17].

Recent work has demonstrated the importance of uncertainty quantification in spatial proteomics [18–20]. [18] proposed a generative classification model and took a Bayesian approach to spatial proteomics data analysis by computing probability distributions of protein-organelle assignments using Markov-chain Monte-Carlo (MCMC). These probabilities were then used as the basis for organelle allocations, as well as to quantify the uncertainty in these allocations. On the basis that some proteins cannot be well described by any of the annotated sub-cellular niches, a multivariate Student’s T distribution was included in the model to enable outlier detection. The proposed T-Augmented Gaussian Mixture (TAGM) model was shown to achieve state-of-the-art predictive performance against other commonly used machine learning algorithms [18]. Furthermore, the model has been successfully applied to reveal unrivalled insight into the spatial organisation of *Toxoplasma gondii* [12] and identify cargo of the Golgins of the trans-Golgi network [21].

Here, we propose an extension to TAGM to allow simultaneous protein-organelle assignments and novelty detection. One assumption of the existing TAGM model is that the number of sub-cellular niches is known. Here, we design a novelty detection algorithm based on allowing an unknown number of additional sub-cellular niches, as well as quantifying uncertainty in this number.

**Fig 1:**
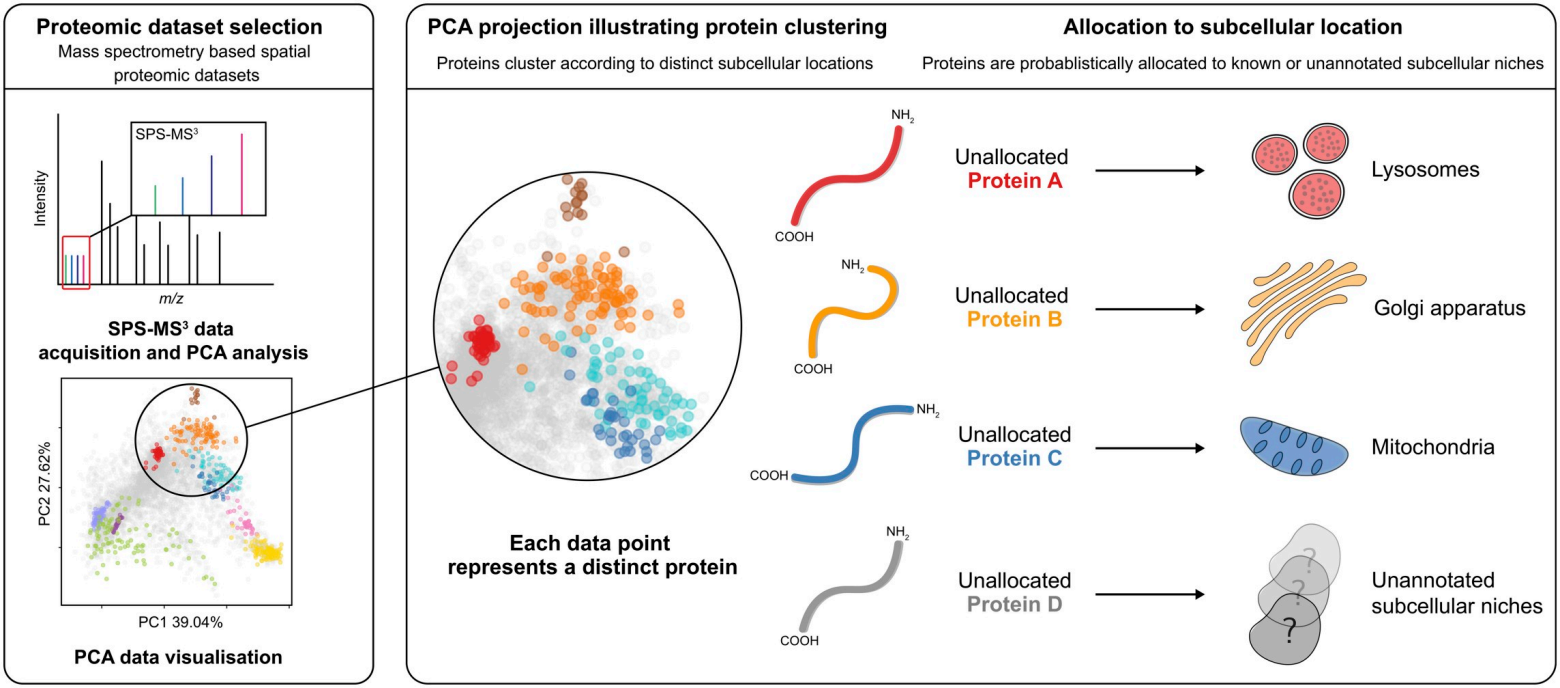
An overview of novelty detection in subcellular proteomics.

Quantifying uncertainty in the number of clusters in a Bayesian mixture model is challenging and many approaches have been proposed in the literature (see for example [22–24] and the appendix for further details). Here, we make use of asymptotic results in Bayesian analysis of mixture models [25]. The principle of *overfitted mixtures* allows us to specify a (possibly large) maximum number of clusters. As shown in [25] these components empty if they are not supported by the data, allowing the number of clusters to be inferred. [26] previously made use of this approach in the Bayesian integrative modelling of multiple genomic datasets. In our application, some of the organelles may be annotated with known marker proteins and this places a lower bound on the number of sub-cellular niches. Bringing these ideas together results in a semi-supervised Bayesian approach, which we refer to as Novelty TAGM (Fig 1. Table 1 summarises the differences between the current available machine-learning methods for spatial proteomics.

**Table 1:** Examples of computational methods for spatial proteomics datasets for prediction and novelty detection.

MS-based Spatial Proteomics Computational Methods for Prediction and Novelty Detection							
Method	Localisation prediction	Uncertainty in protein localisation	Outlier detection	Novelty detection	Uncertainty in number of novel phenotypes	Uncertainty in allocation to new phenotypes	Integrative
Supervised Machine Learning (as reviewed in [11])	✓	✘	✘	✘	✘	✘	✘
Correlation Profiling [29, 30]	✓	✘	✘	✘	✘	✘	✘
Transfer Learning [31]	✓	✘	✘	✘	✘	✘	✓
*Mclust* (as used in [9])	✘	✘	✓	✓	✘	✘	✘
*PhenoDisco* [13]	✘	✘	✓	✓	✘	✘	✘
TAGM [18]	✓	✓	✓	✘	✘	✘	✘
Novelty TAGM (This manuscript)	✓	✓	✓	✓	✓	✓	✘

We apply Novelty TAGM to 10 spatial proteomic datasets across a diverse range of protocols, including *hyper*LOPIT [4, 7], LOPIT-DC [8], Dynamic Organellar Maps (DOM) [27] and spatial-temporal methods [28]. Application of Novelty TAGM to each dataset reveals additional biologically relevant compartments. Notably, we detect 4 sub-nuclear compartments in the the U-2 OS *hyper*LOPIT dataset: the nucleolus, nucleoplasm, chromatin-associated, and the nuclear membrane. In addition, an endosomal compartment is robustly identified across *hyper*LOPIT and LOPIT-DC datasets. Finally, we also uncover collections of proteins with previously uncharacterised localisation patterns; for example, vesicle proteins trafficking from the ER to the early Golgi in *Saccharomyces cerevisiae*.

## Methods

### Datasets

We provide a brief description of the datasets used in this manuscript. We analyse *hyper*LOPIT data, in which sub-cellular fractionation is performed using density-gradient centrifugation [7, 15, 32], on pluripotent mESCs (E14TG2a) [4], human bone osteosarcoma (U-2 OS) cells [5, 8], and *S. cerevisiae* (baker’s yeast) cells [33]. The mESC dataset combines two 10-plex biological replicates and quantitative information on 5032 proteins. The U-2 OS dataset combines three 20-plex biological replicates and provides information on 4883 proteins. The yeast dataset represents four 10-plex biological replicate experiments performed on *S. cerevisiae* cultured to early-mid exponential phase. This dataset contains quantitative information for 2846 proteins that were common across all replicates. Tandem Mass Tag (TMT) [34] labelling was used in all *hyper*LOPIT experiments with LC-SPS-MS^3^ used for high accuracy quantitation [35, 36]. [28] integrated a temporal component to the LOPIT protocol. They analysed HCMV-infected primary fibroblast cells over 5 days, producing control and infected maps every 24 hours. We analyse the control and infected maps 24 hours post-infection, providing information on 2220 and 2196 proteins respectively. In a comparison with *phenoDisco*, we apply Novelty TAGM to a dataset acquired using LOPIT-based fractionation and 8-plex iTRAQ labelling on the HEK-293 human embryonic kidney cell line, quantifying 1371 proteins [13].

Our approach is not limited to spatial proteomics data where the sub-cellular fractionation is performed using density gradients. We demonstrate this through the analysis of DOM datasets on HeLa cells and mouse primary neurons [27, 37], which quantify 3766 and 8985 proteins respectively. These approaches used SILAC quantitation with differential centrifugation-based fractionation. We analyse 6 replicates from the HeLa cell line analyses in [27] and 3 replicates from the mouse primary neuron experiments in [37]. [38] also used the DOM protocol coupled with CRISPR-CAS9 knockouts in order to explore the functional role of AP-5. We analyse the control map from this experiment. Finally, we consider the U-2 OS data which were acquired using the LOPIT-DC protocol [8] and quantified 6837 proteins across 3 biological replicates. In favour of brevity, we do not consider protein correlation profiling (PCP) based spatial proteomics datasets in this manuscript, though our method also applies to such data [29, 39, 40] and other sub-cellular proteomics methods which utilised cellular fractionation [9].

### Model

#### Spatial proteomics mixture model

In this section, we briefly review the TAGM model proposed by [18]. Let *N* denote the number of observed protein profiles each of length *L*, corresponding to the number of quantified fractions. The quantitative profile for the *i*-th protein is denoted by **x**_*i*_ = [*x*_1*i*_,…,*x*_*Li*_]. In the original formulation of the model it is supposed that there are *K* known sub-cellular compartments to which each protein could be localised (e.g. cytosol, endoplasmic reticulum, mitochondria, …). For simplicity of exposition, we refer to these *K* sub-cellular compartments as *components*, and introduce component labels *z*_*i*_, so that *z*_*i*_ = *k* if the *i*-th protein localises to the *k*-th component. To fix notation, we denote by *X*_*L*_ the set of proteins whose component labels are known, and by *X*_*U*_ the set of unlabelled proteins. If protein *i* is in *X*_*U*_, we seek to evaluate the probability that *z*_*i*_ = *k* for each *k* = 1, …, *K*; that is, for each unlabelled protein, we seek the probability of belonging to each component (given a model and the observed data).

The distribution of quantitative profiles associated with each protein that localises to the *k*-th component is modelled as multivariate normal with mean vector ***μ***_*k*_ and covariance matrix Σ_*k*_. However, many proteins are dispersed and do not fit this assumption. To model these “outliers”, [18] introduced a further indicator variable *ϕ*. Each protein **x**_*i*_ is then described by an additional variable *ϕ*_*i*_, with *ϕ*_*i*_ = 1 indicating that protein **x**_*i*_ belongs to an organelle-derived component and *ϕ*_*i*_ = 0 indicating that protein **x**_*i*_ is not well described by these known components. This *outlier component* is then modelled as a multivariate T distribution with degrees of freedom *κ*, mean vector **M**, and scale matrix *V*. Thus the model can be written as:

$$\begin{array}{c} {\text{x}}_{i}|z_{i}=k,\phi_{i}\;∼N{\left(\mu_{k},\Sigma_{k}\right)}^{\phi_{i}}T{\left(\kappa ,M,V\right)}^{1-\phi_{i}}. \end{array}$$ (1)

Let *f*(**x**|***μ***, Σ) denote the density of the multivariate normal with mean vector ***μ*** and covariance matrix Σ evaluated at **x**, and similarly let *g*(**x**|*κ*, **M**, **V**) denote the density of the multivariate T-distribution. For any *i*, the prior probability of the *i*-th protein localising to the *k*-th component is denoted by *p*(*z*_*i*_ = *k*) = *π*_*k*_. Letting $\theta ={\left\{\mu_{k},\Sigma_{k}\right\}}_{k=1}^{K}$ denote the set of all component mean and covariance parameters, and $\pi ={\left\{\pi_{k}\right\}}_{k=1}^{K}$ denote the set of all mixture weights, it follows that:

$$\begin{array}{c} \begin{array}{cc} p({\text{x}}_{i}|\theta ,\pi ,\phi_{i},\kappa ,\text{M},V) & =\sum_{k=1}^{K}\pi_{k}(f{\left({\text{x}}_{i}|\mu_{k},\Sigma_{k}\right)}^{\phi_{i}}g{\left({\text{x}}_{i}|\kappa ,M,V\right)}^{1-\phi_{i}}). \end{array} \end{array}$$ (2)

For any *i*, we set the prior probability of the *i*-th protein belonging to the outlier component as *p*(*ϕ*_*i*_ = 0) = *ϵ*, where *ϵ* is a parameter that we infer.

Eq (2) can then be rewritten in the following way:

$$\begin{array}{c} \begin{array}{cc} p({\text{x}}_{i}|\theta ,\pi ,\kappa ,\epsilon ,\text{M},V) & =\sum_{k=1}^{K}\pi_{k}(\left(1-\epsilon \right)(f\left({\text{x}}_{i}|\mu_{k},\Sigma_{k}\right)+\epsilon g\left({\text{x}}_{i}|\kappa ,M,V\right)), \end{array} \end{array}$$ (3)

As in [18], we fix *κ* = 4, **M** as the global empirical mean, and *V* as half the global empirical variance of the data, including labelled and unlabelled proteins. To extend this model to permit novelty detection, we specify the maximum number of components *K*_*max*_ > *K*. Our proposed model then allows up to *K*_*novelty*_ = *K*_*max*_ − *K* ≥ 0, new phenotypes to be detected. Eq 3 can then be written as

$$\begin{array}{c} \begin{array}{cc} p\left({\text{x}}_{i}|\theta ,\pi ,\kappa ,\epsilon ,\text{M},V\right)=\sum_{k=1}^{K} & \pi_{k}(\left(1-\epsilon \right)(f\left({\text{x}}_{i}|\mu_{k},\Sigma_{k}\right)+\epsilon g\left({\text{x}}_{i}|\kappa ,M,V\right)))\\ + & \sum_{k=K+1}^{K_{max}}\pi_{k}(\left(1-\epsilon \right)(f\left({\text{x}}_{i}|\mu_{k},\Sigma_{k}\right)+\epsilon g\left({\text{x}}_{i}|\kappa ,M,V\right))), \end{array} \end{array}$$ (4)

where, in the first summation, the *K* components correspond to known sub-cellular niches and the second summation corresponds to the new phenotypes to be inferred. The parameter sets are then augmented to include these possibly new components; that is, we redefine $\theta ={\left\{\mu_{k},\Sigma_{k}\right\}}_{k=1}^{K_{max}}$ to denote the set of all component mean and covariance parameters, and $\pi ={\left\{\pi_{k}\right\}}_{k=1}^{K_{max}}$ denotes the set of all mixture weights. Relying on the principle of over-fitted mixtures [25], components that are not supported by the data are left empty with no proteins allocated to them. We find setting *K*_*novelty*_ = 10 is ample to detect new phenotypes. To complete our Bayesian model, we need to specify priors. Detailed prior specifications and sensitivity analysis are provided in the S1 Text.

#### Bayesian inference and convergence

We perform Bayesian inference using Markov-chain Monte-Carlo methods. We make modifications to the collapsed Gibbs sampler approach used previously in [18] to allow inference to be performed for the parameters of the novel components (see S1 Text for full details). Since the number of occupied components at each iteration is random, we monitor this quantity as a convergence diagnostic.

#### Visualising patterns in uncertainty

To simultaneously visualise the uncertainty in the number of newly discovered phenotypes, as well as the uncertainty in the allocation of proteins to new components, we use the so-called *posterior similarity matrix* (PSM) [41]. The PSM is an *N* × *N* matrix where the (*i*, *j*)^*th*^ entry is the posterior probability that protein *i* and protein *j* reside in the same component. Throughout we use a heatmap representation of this quantity. The PSM is summarised into a clustering by maximising the posterior expected adjusted Rand index (see appendix for details; [41]). Formulating inference around the PSM also avoids some technical statistical challenges, which are discussed in detail in the appendix.

#### Uncertainty quantification

We may be interested in quantifying the uncertainty in whether a protein belongs to a new sub-cellular component. Indeed, it is important to distinguish whether a protein belongs to a new phenotype or if we simply have large uncertainty about its localisation. The probability that protein *i* belongs to a new component is computed from the following equation:

$$P(z_{i}\in \left\{K+1,\ldots ,K_{max}\right\}|X)=1-P(z_{i}\in \left\{1,\ldots ,K\right\}|X),$$ (5)

which we approximate by the following Monte-Carlo average:

$$\begin{array}{c} 1-\frac{1}{T}\sum_{t=1}^{T}P\left(z_{i}^{\left(t\right)}\in \left\{1,\ldots ,K\right\}|X\right)=1-\frac{1}{T}\sum_{t=1}^{T}\sum_{k=1}^{K}P\left(z_{i}^{\left(t\right)}=k|X\right), \end{array}$$ (6)

where *T* is the number of Monte-Carlo iterations. Throughout, we refer to Eq 6 as the *discovery probability*.

#### Applying the model in practice

Applying Novelty TAGM to spatial proteomics datasets consists of several steps. After having run the algorithm on a dataset and assessing convergence, we proceed to explore the ouput of the method. We explore *putative phenotypes*, which we define as newly discovered clusters with at least 1 protein with discovery probability greater than 0.95.

### Validating computational approaches

In a supervised framework the performance of computational methods can be assessed by using the training data, where a proportion of the training data is withheld from the classifier to be used for the assessment of predictive performance. In an unsupervised or semi-supervised framework we cannot validate in this way, since there is no “ground truth” with which to compare. Thus, we propose several approaches, using external information, for validation of our method.

#### Artificial masking of annotations to recover experimental design

Removing the labels from an entire component and assessing the ability of our method to rediscover these labels is one form of validation. We consider this approach for several of the datasets; in particular, chromatin enrichment was performed in two of the *hyper*LOPIT experiments, where the intention was to increase the resolution between chromatin and non-chromatin associated nuclear proteins [4, 5, 7]. As validation of our method we hide these labels and seek to rediscover them in an unbiased fashion.

#### The Human Protein Atlas

A further approach to validating our method is to use additional spatial proteomic information. The Human Protein Atlas (HPA) [5, 42] provides confocal microscopy information on thousands of proteins, using validated antibodies. When we consider a dataset for which there is HPA annotation, we use this data to validate the novel phenotypes for biological relevance.

#### Gene Ontology (GO) term enrichment

Throughout, we perform GO enrichment analysis with FDR control performed according to the Benjamini-Höchberg procedure [43–45]. The proteins in each novel putative phenotype are assessed in turn for enriched Cellular Component terms, against the background of all quantified proteins in that experiment.

#### Robustness across multiple MS-based spatial proteomics datasets

On occasion some cell lines have been analysed using multiple spatial proteomics technologies [8]. In these cases, the putative phenotypes discovered by Novelty TAGM are compared directly. If the same phenotype is discovered in different proteomic datasets we consider this as robust evidence for sufficient resolution of that phenotype.

## Results

Motivated by the need for novelty detection methods which also quantify the uncertainty in the number of clusters and the assignments of proteins to each cluster, we developed Novelty TAGM (see Methods). This approach extends our previous TAGM model [18] to enable the detection of novel *putative phenotypes*, which we define as newly discovered clusters with at least 1 protein with discovery probability greater than 0.95. Our proposed methodology allows us to interrogate individual proteins to assess whether they belong to a newly discovered phenotype. Through the *posterior similarity matrix* (PSM) we can visualise the global patterns in the uncertainty in phenotype discovery (see supplement). We summarise this posterior similarity matrix into a single clustering by maximising the posterior expected adjusted rand index (see Methods). This methodology infers the number of clusters supported by the data, in contrast to many existing approaches which require specification of the number of clusters (such as K-means or Mclust [46]). To demonstrate the value of this approach, we applied Novelty TAGM to a diverse set of spatial proteomics datasets.

### Validating experimental design in *hyper*LOPIT

Initially, we validated Novelty TAGM in a setting where we have a strong *a priori* expectation for the presence of an unannotated niche. For this we used a human bone osteosarcoma cell (U-2 OS) *hyper*LOPIT dataset [5] and an mESC *hyper*LOPIT dataset [4]. These experimental protocols used a chromatin enrichment step to resolve nuclear chromatin-associated proteins from nuclear proteins not associated with chromatin. Removing the nuclear, chromatin and ribosomal annotations from the datasets, we test the ability of Novelty TAGM to recover them.

#### Human bone osteosarcoma (U-2 OS) cells

**Fig 2:**
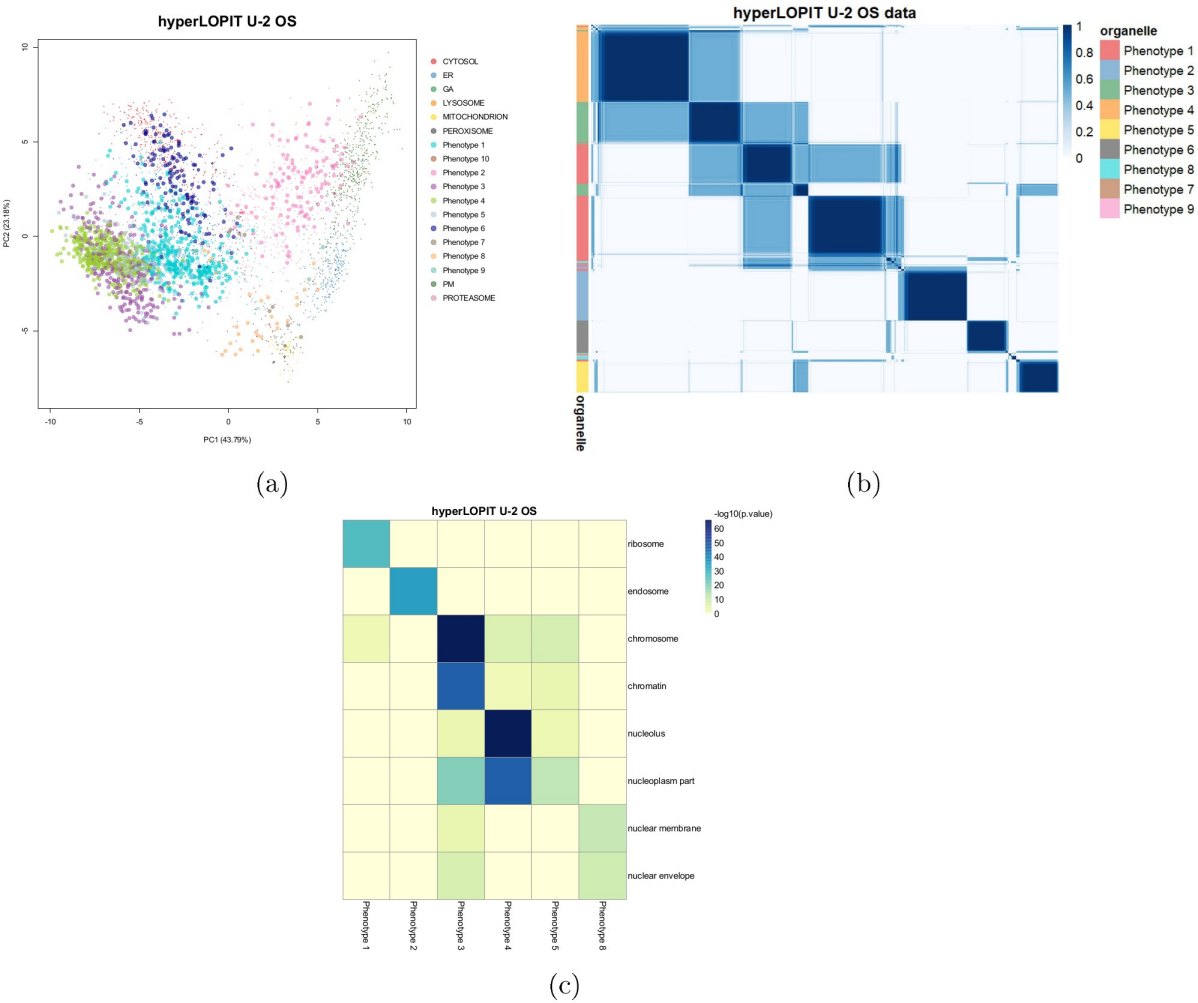
(a) PCA plot of the *hyper*LOPIT U-2 OS cancer cell line data. Points are scaled according to the discovery probability with larger points indicating greater discovery probability. (b) Heatmaps of the posterior similarity matrix derived from U-2 OS cell line data demonstrating the uncertainty in the clustering structure of the data. We have only plotted the proteins which have greater than 0.99 probability of belonging to a new phenotype and probability of being an outlier less than 0.5 for the U-2 OS dataset to reduce the number of visualised proteins. (c) Tile plot of discovered phenotypes against GO CC terms to demonstrate over-representation, where the colour intensity is the -log_10_ of the p-value.

For the U-2 OS dataset, Novelty TAGM reveals 9 putative phenotypes, which we refer to as phenotype 1, phenotype 2, etc… These phenotypes, along with the uncertainty associated with them, are visualised in Fig 2. We consider the HPA confocal microscopy data for validation [5, 42]. The HPA provides information on the same cell line and therefore constitutes an excellent complementary resource. This *hyper*LOPIT dataset was already shown to be in strong agreement with the microscopy data [5, 8]. Proteins in phenotypes 3, 4, 5 and 8 have a nucleus-related annotation as their most frequent HPA annotation, as well as differential enrichment of nucleus-related GO terms (Fig 2). Phenotype 3 validates the chromatin enrichment preparation (Fig 2C) and phenotype 4 reveals a nucleoli cluster, where nucleoli and nucleoli/nucleus are the 2^*nd*^ and 3^*rd*^ most frequent HPA annotations for proteins belonging to this phenotype. For phenotype 5, the most associated term is nucleoplasm from the HPA data and this is further supported by GO analysis (Fig 2C). Phenotype 8 demonstrates further sub-nuclear resolution and has *nuclear membrane* as its most frequent HPA annotation and has corresponding enriched GO terms (Fig 2C). In addition, phenotypes 1 and 2 are enriched for *ribosomes* and *endosomes* respectively.

#### Pluripotent mESCs (E14TG2a)

In the case of the mESC dataset, Novelty TAGM reveals 8 new putative phenotypes. The chromatin enrichment preparation is also validated in these cells, as well as new phenotypes with additional annotations such *nucleolus* and *centrosome* (see S1 Text). We also used this dataset to explore how our results are impacted if we reduce the number of markers from other niches (see S1 Text).

### Uncovering additional sub-cellular structures

Having validated the ability of Novelty TAGM to recover known experimental design, as well as uncover additional sub-cellular niches resolved in the data, we turn to apply Novelty TAGM to several additional datasets.

#### U-2 OS cell line revisited

**Fig 3:**
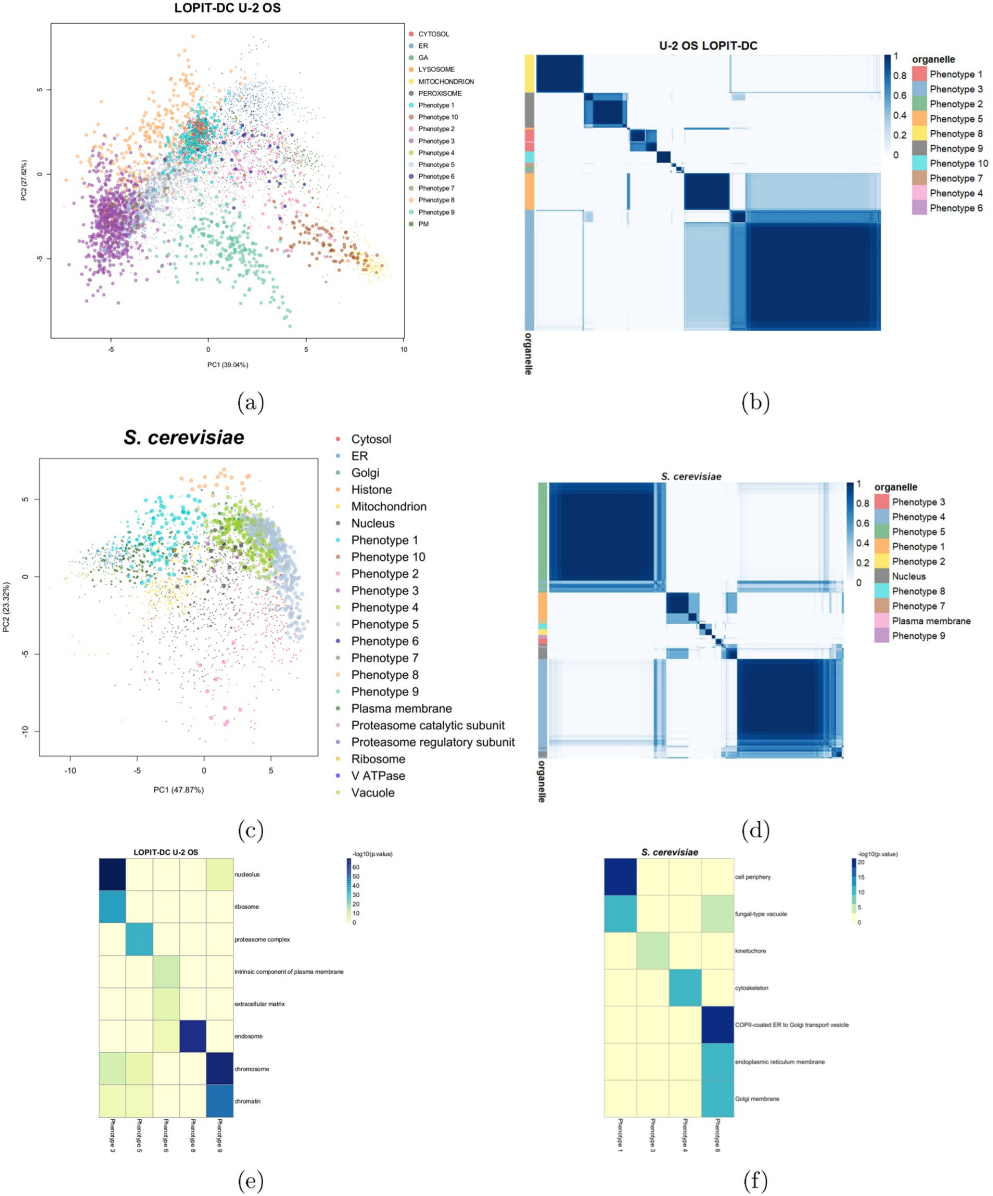
(a, c) PCA plots of the LOPIT-DC U-2 OS data and the *hyper* LOPIT yeast data. The points are scaled according to the discovery probability. (b, d) Heatmaps of the posterior similarity matrix derived from the U-2 OS and yeast datasets demonstrating the uncertainty in the clustering structure of the data. We have only plotted the proteins which have greater than 0.99 probability of belonging to a new phenotype and probability of being an outlier less than 0.95 (10^−5^ for LOPIT-DC to reduce the number of visualised proteins). (e, f) Tile plots of phenotypes against GO CC terms where the colour intensity is the -log_10_ of the p-value.

We first consider the LOPIT-DC dataset on the U-2 OS cell line [8]. Again, we removed the nuclear, proteasomal, and ribosomal annotations. Novelty TAGM reveals 10 putative phenotypes (Fig 3).

In a similar vein to the analysis performed on the *hyper*LOPIT U-2 OS dataset, we initially use the available HPA data to validate these clusters [5]. Phenotypes 3, 5, 7 and 9 display nucleus-associated terms as their most frequent HPA annotation. Clear differential enrichment of phenotypes with GO Cellular Component terms is evident from Fig 3E. This analysis reveals *nucleolus*, *ribosome*, *proteasome* phenotypes. Furthermore, a *chromatin* phenotype is also resolved. Notably, this is the first evidence for sub-nuclear resolution in this LOPIT-DC dataset. Phenotype 6 represents a cluster with mixed *plasma membrane* and *extracellular matrix* annotations and this is supported by HPA annotation with vesicles, cytosol, and plasma membrane being the top three annotations. An extracellular matrix-related phenotype was not previously known in these data and might correspond to exocytic vesicles containing ECM proteins. Furthermore, phenotype 8 is significantly enriched for *endosomes*, again a novel annotation for this data. In addition, 107 of the proteins in this phenotype are also localised to the endosome-enriched phenotype presented in the U-2 OS *hyper*LOPIT dataset (section Human bone osteosarcoma (U-2 OS) cells). Thus, we robustly identify new phenotypes across different spatial proteomics protocols. Hence, we have presented strong evidence for additional annotations in this dataset, beyond the original analysis of the data [8]. In particular, although a separate chromatin enrichment preparation was not included in the U-2 OS LOPIT-DC analysis and the original authors did not identify sufficient resolution between the nucleus and chromatin clusters in this dataset, Novelty TAGM could, in fact, reveal a chromatin-associated phenotype in the U-2 OS LOPIT-DC data. In addition, we have joint evidence for an endosomal cluster in both the LOPIT-DC and *hyper*LOPIT datasets. Finally, through the discovery probability and by using the PSMs we have quantified uncertainty in these proposed phenotypes, enabling more rigorous interrogation of these datasets.

#### Saccharomyces cerevisiae

Novelty TAGM uncovers 8 putative phenotypes in the yeast *hyper*LOPIT data [33]. Four of these phenotypes have no significant over-represented annotations. Fig 3F demonstrates that the remaining four phenotypes are differentially enriched for GO terms. Firstly, a mixed *cell periphery* and *fungal-type* vacuole phenotype is uncovered along with a *kinetochore* phenotype, and a *cytoskeleton* phenotype. Phenotype 8 represents a joint Golgi and ER cluster with several enriched GO terms. Indeed, most of the proteins in this phenotype have roles in the early secretory pathway that involve either transport from the ER to the early Golgi apparatus, or retrograde transport from the Golgi to the ER [47–50], (also reviewed in [51]). To be precise, 11 out of the total 20 proteins in this cluster are annotated as core components of COPII vesicles and 6 associated with COPI vesicles. The protein Ksh1p (Q8TGJ3) is further suggested through homology with higher organisms to be part of the early secretory pathway [52]. The proteins Scw4p (P53334), Cts1p (P29029) and Scw10p (Q04951) [53], as well as Pst1p (Q12355) [54], and Cwp1p (P28319) [55], however, are annotated in the literature as localising to the cell wall or extracellular region. It is therefore possible that their predicted co-localisation with secretory pathway proteins observed here reflects a proportion of their lifecycle being synthesised or spent trafficking through the secretory pathway. The protein Ssp120p (P39931) is of unknown function and has been shown to localise in high throughput studies to the vacuole [50] and to the cytoplasm in a punctate pattern [56]. The localisation observed here may suggest that it is therefore either part of the secretory pathway, or trafficks through the secretory organelles for secretion or to become a constituent of the cell wall.

#### Fibroblast cells

We also uncover additional annotations for the HCMV infected and the control fibroblast spatial proteomics datasets [28]; such as, sub-mitochondrial annotations, as well as resolution of the small and large ribosomal sub-units. These annotations were overlooked in the original analysis [28] and further details can be found in the S1 Text.

### Refining annotation in organellar maps

The Dynamic Organellar Maps (DOM) protocol was developed as a faster method for MS-based spatial proteomic mapping, albeit at the cost of lower organelle resolution [27, 57]. The three datasets analysed here are two HeLa cell lines [27, 38] and a mouse primary neuron dataset [37]. All three of these datasets have been annotated with a class called “large protein complexes”. This class contains a mixture of cytosolic, ribosomal, proteasomal and nuclear sub-compartments that pellet during the centrifugation step used to capture this mixed fraction [27]. We apply Novelty TAGM to these data and remove this “large protein complexes” class, to derive more precise annotations for these datasets.

#### HeLa cells (Itzhak et. al 2016)

**Fig 4:**
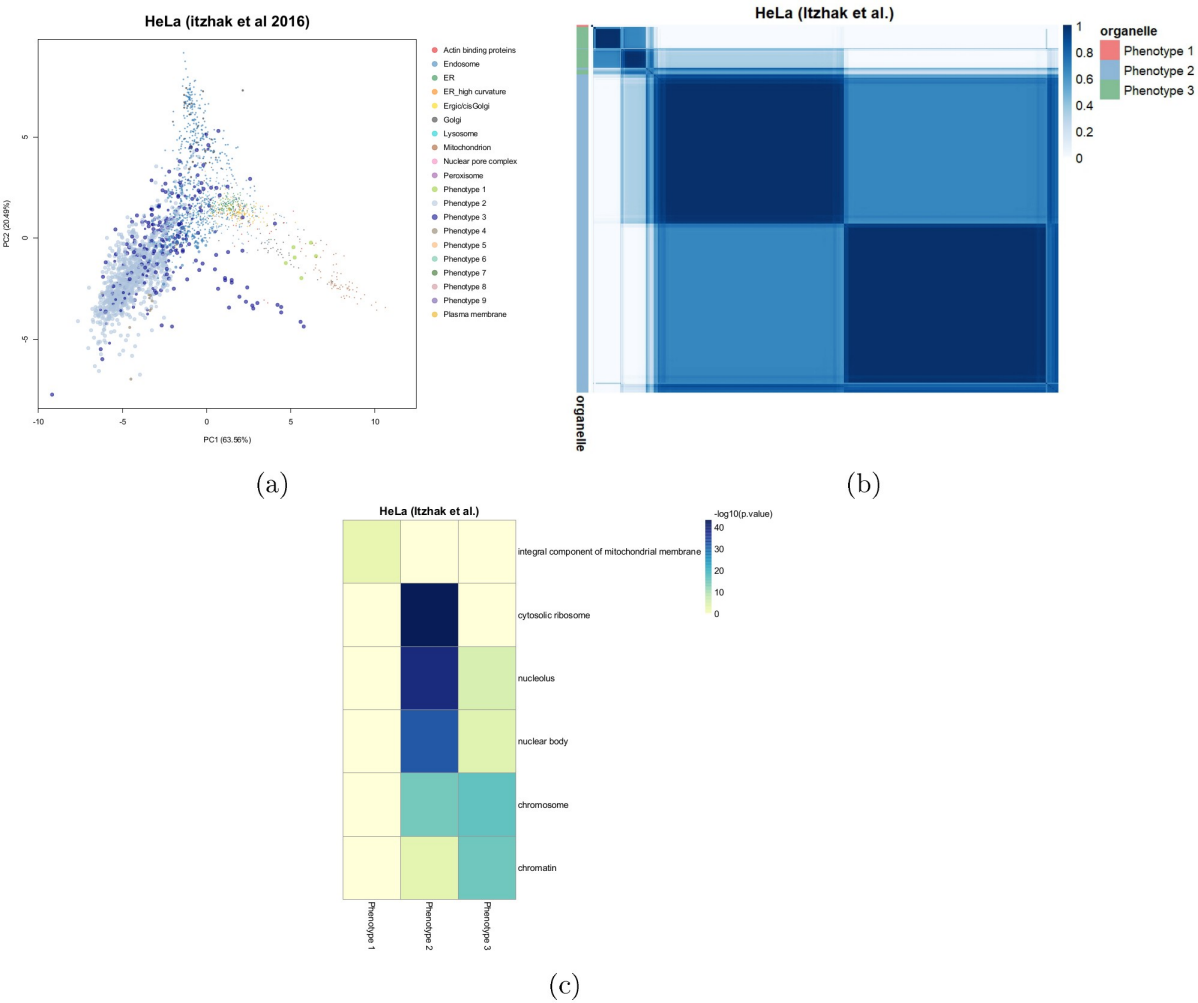
(a) PCA plots of the HeLa data. The pointers are scaled according to their discovery probability. (b) Heatmaps of the HeLa Itzhak data. Only the proteins with discovery probability greater than 0.99 and outlier probability less than 0.95 are shown. The heatmaps demonstrate the uncertainty in the clustering structure present in the data. (c) Tile plot of phenotypes against GO CC terms where the colour intensity is the -log_10_ of the p-value.

The HeLa dataset of [27] has 3 additional phenotypes uncovered by Novelty TAGM. Fig 4C shows a *mitochondrial membrane* phenotype, distinct from the already annotated mitochondrial class. Phenotype 2 represents a mixed cluster with nucleus-, ribosome- and cytosol-related enriched terms. The final phenotype is enriched for *chromatin* and *chromosome*, suggesting sub-nuclear resolution. Furthermore, as a result of quantifying uncertainty, we can see that there are potentially more sub-cellular structures in this data (Fig 4). However, the uncertainty is too great to support these phenotypes.

#### Mouse primary neurons and HeLa cells (Hirst et. al 2018)

Application of Novelty TAGM to mouse primary neuron data [37] and another HeLa dataset [38] yields further annotations; such as, *ribosomal*, *cytosolic* and *extracellular* annotations (see S1 Text).

## Comparison between Novelty TAGM and *phenoDisco*

Next, we compare an already available novelty detection algorithm, *phenoDisco*, with Novelty TAGM. Despite both methods performing novelty detection, the algorithms are quite distinct. The first major difference is that Novelty TAGM is a Bayesian method that performs uncertainty quantification. Novelty TAGM quantifies the uncertainty in both the number of newly identified phenotypes and whether individual proteins should belong to a new phenotype. On the other hand, *phenoDisco* uses the *Bayesian Information Criterion* (BIC) to select just a single clustering, without taking into account the uncertainty in the number of phenotypes, and does not provide an estimate of individual protein-to-phenotype allocation uncertainty. Another difference is the input to both methods; Novelty TAGM uses the data directly, whereas *phenoDisco* takes the top principal components (by default, the first two) as input. *PhenoDisco* also requires an additional parameter—the minimum group size. This parameter can be challenging to specify, since there is a trade-off between identifying functionally relevant phenotypes of different sizes and picking up small spurious protein clusters. Furthermore, *phenoDisco* struggles to scale to many of the datasets presented in this manuscript, because it requires iteratively refitting models and building of an outlier test statistic.

**Fig 5:**
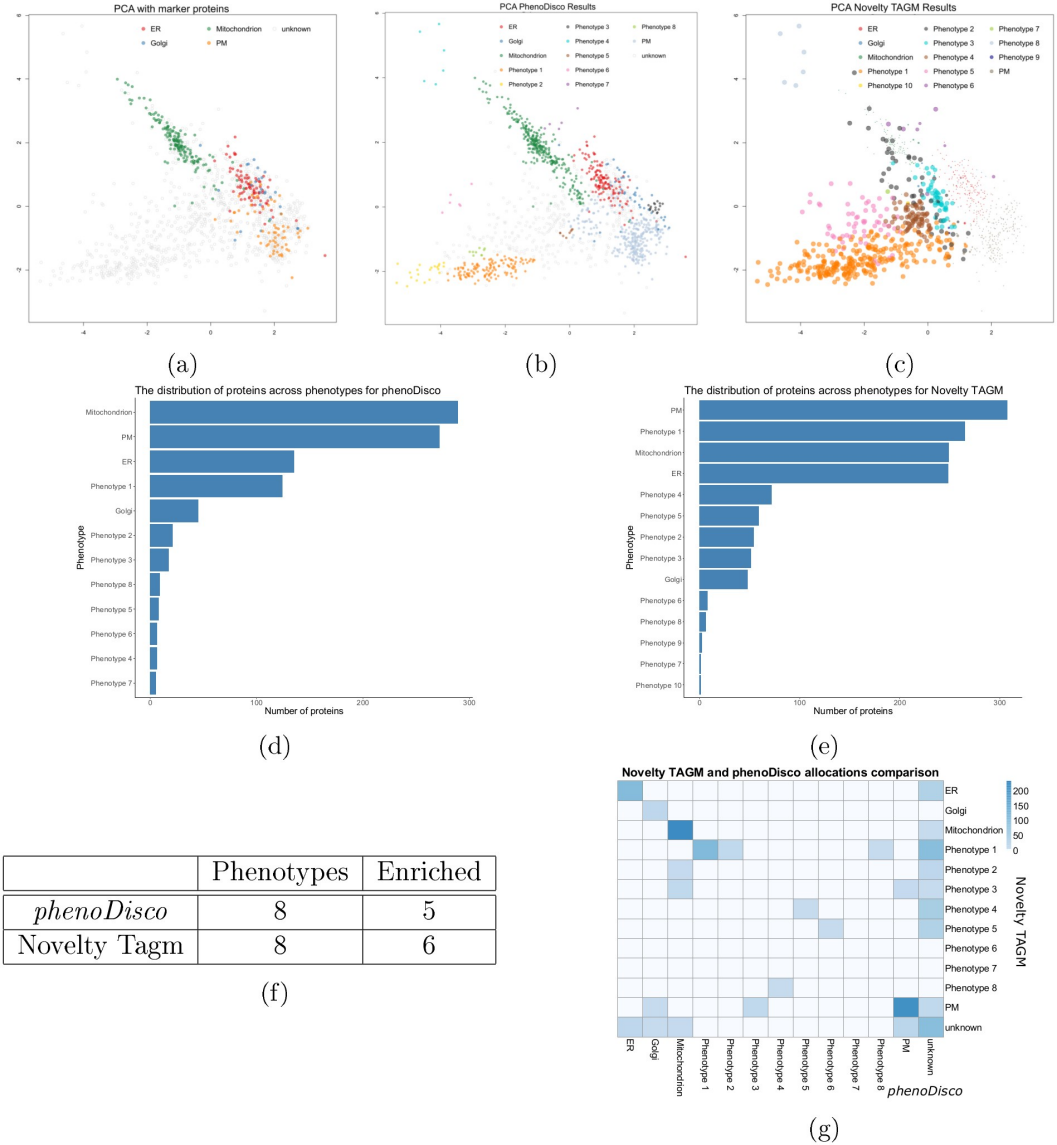
(a) PCA plot showing marker proteins for the HEK-293 dataset. (b) PCA plot with phenotypes identified by *phenoDisco*. (c) PCA plot with phenotypes identified by Novelty TAGM with pointer size scaled to discovery probability. (d, e) Barplots showing the number of proteins allocated to different phenotypes by *phenoDisco* and Novelty TAGM respectively. (f) A table demonstrating the number of phenotypes with functional enrichment for both methods and the number of phenotypes discovered. (g) A heatmap showing the overlap between *phenoDisco* and Novelty TAGM allocations.

To demonstrate the differences between the two approaches, we apply *phenoDisco* and Novelty TAGM to the HEK-293 spatial proteomics dataset interrogated by [13]. The PCA plots in Fig 5 reveal broad similarities in the location of the discovered phenotypes. Novelty TAGM provides more information than *phenoDisco*; for example, we can scale the pointer size to the discovery probability. We note that both methods reveal 8 putative phenotypes in the data. Fig 5D and 5E reveals the distribution of proteins across these phenotypes. We conclude that both approaches are able to discover small and large clusters, with both methods identifying phenotypes with a few proteins, but also phenotypes with greater than 100 proteins. Fig 5F shows that both methods find the same number of phenotypes; however, not all of these phenotypes are functionally enriched. For *phenoDisco*, four of the phenotypes had at least 1 significant Gene Ontology term, whereas this was true for five of the Novelty TAGM phenotypes. Fig 5G characterises the protein overlap between the two approaches. We see that both methods are in broad agreement, with most of the disagreement attributed to cases where one method assigns a protein as unknown whilst the other allocates to it a phenotype or organelle. For example, Novelty TAGM associates *phenoDisco* phenotype 3, which is a lysosome-enriched phenotype, with the plasma membrane (albeit with low probability). On the other hand, Novelty TAGM phenotypes 2 and 3, enriched for chromatin and ribosome respectively, are associated with the mitochondria by *phenoDisco*. This demonstrates the ability of Novelty TAGM to derive more biologically meaningful phenotypes.

## Improved annotation allows exploration of endosomal processes

**Fig 6:**
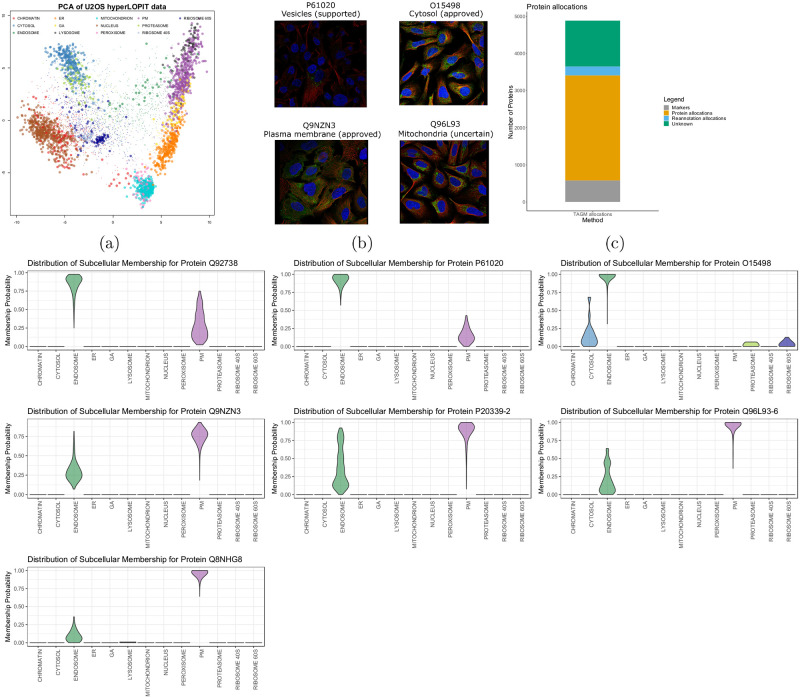
(a) PCA of U-2 OS *hyper*LOPIT data with pointer scaled to localisation probability and outliers shrunk. Points are coloured according to their most probable organelle. (b) Immunofluorescence images and sub-cellular localisation annotation taken from the HPA database (https://www.proteinatlas.org/humanproteome/cell) for the proteins with UniProt accessions P61020 (Rab5b), O15498 (Ykt6), Q9NZN3 (EHD3), and Q96L93 (KIF16B). The nucleus is stained in blue; microtubules in red, and the antibody staining targeting the protein in green. (c) A barplot representing the number of proteins allocated before and after re-annotation of the endosomal class. (d) Violin plots of full probability distribution of proteins to organelles, where each violin plot is for a single protein.

Given the information that the U-2 OS *hyper*LOPIT dataset resolves an endosomal cluster not previously explored, we perform a re-analysis of this dataset focusing on the endosomes. We curate a set of marker proteins for the endosomes and add these annotations to the U-2 OS *hyper*LOPIT dataset. After which, we apply our Bayesian generative classifier TAGM to the data with this additional annotation [18]. Protein allocations to each sub-cellular niche are visualised in the PCA plot of Fig 6A. Fig 6C demonstrates the increased number of proteins that can be characterised by improved annotation of the U-2 OS cell dataset. Furthermore, we examine 7 (of 240) proteins with uncertain endosomal localisation, which can be visualised in each of the violin plots in Fig 6D.

All 7 proteins with uncertain assignment to our new endosome cluster are known to function in endosome dynamics. Rab5a and Rab5b (P20339; P61020) are isoforms of Rab5, a small GTPase which is considered a master organiser of the endocytic system, regulating clathrin-mediated endocytosis and early endosome dynamics [58–65]. RN-tre (Q92738) is a GTPase-activating protein which controls the activity of several Rab GTPases, including Rab5, and is therefore a key player in the organisation and dynamics of the endocytic pathway [64, 66]. KIF16B (Q96L93) is a plus end-directed molecular motor which regulates early endosome motility along microtubules. It is required for the establishment of the steady-state sub-cellular distribution of early endosomes, as well as the balance between PM recycling and lysosome degradation of signal transducing cell surface receptors including EGFR and TfR [67, 68]. Notably, it has been demonstrated that KIF16B co-localises with the small GTPase Rab5, whose isoforms Rab5a and Rab5b we also identified as potentially localised to the endosome and PM in this dataset. ZNRF2 (Q8NHG8) is an E3 ubiquitin ligase which has been shown to regulate mTOR signalling as well as lysosomal acidity and homeostasis in mouse and human cells and has been detected at the endosomes, lysosomes, Golgi apparatus and PM according to the literature [69, 70]. Ykt6 (O15498) is a SNARE (soluble N-ethylmaleimide-sensitive factor attachment protein receptor) protein that regulates a wide variety of intracellular trafficking and membrane tethering and fusion processes. The membrane-associated form of Ykt6 has been detected at the PM, ER, Golgi apparatus, endosomes, lysosomes, vacuoles (in yeast), and autophagosomes as part of various SNARE complexes [71–78]. In line with this, our results show a mixed sub-cellular distribution for Ykt6 with potential localisation to the endosome and cytosol (Fig 6D). EHD3 (Q9NZN3) is an important regulator of endocytic trafficking and recycling, which promotes the biogenesis and stabilisation of tubular recycling endosomes by inducing early endosome membrane bending and tubulation [79, 80]. We observe a mixed steady-state potential localisation to the endosome and PM for EHD3 (Fig 6D). This is in agreement with EHD3’s role in recycling endosome-to-PM transport [80–84].

Of these 7 proteins with uncertain endosome assignment, only 4 have localisations annotated in HPA (Fig 6(b)). The HPA assigns Rab5b to the vesicles which, in this context, include the endosomes, lysosomes, peroxisomes and lipid droplets. Therefore, a more precise annotation is available using Novelty TAGM. Ykt6 is localised to the cytosol, in support of our observations. EHD3 has approved localisation to the plasma membrane, again in agreement with our assignments. KIF16B is assigned to the mitochondrion, which contradicts our findings as well as previously published literature on the localisation and biological role of this protein. We speculate that this disagreement arises from the uncertainty associated with the specificity of the chosen antibody [5]. Thus, Novelty TAGM enables sub-cellular fractionation-based methods to identify proteins in sub-cellular niches which can not be fully interrogated by immunocytochemistry.

## Discussion

We have presented a semi-supervised Bayesian approach that simultaneously allows probabilistic allocation of proteins to organelles, detection of outlier proteins, as well as the discovery of novel sub-cellular structures. Our method unifies several approaches present in the literature, combining the ideas of supervised machine learning and unsupervised structure discovery. Formulating inference in a Bayesian framework allows for the quantification of uncertainty; in particular, the uncertainty in the number of newly discovered annotations.

Application of our method across 10 different spatial proteomic datasets acquired using diverse fractionation and MS data acquisition protocols and displaying varying levels of resolution revealed additional annotation in every single dataset. Our analysis recovered the chromatin-associated protein phenotype and validated experimental design for chromatin enrichment in *hyper*LOPIT datasets. Our approach also revealed additional sub-cellular niches in the mESC *hyper*LOPIT and U-2 OS hyperLOPIT datasets.

Our method revealed resolution of 4 sub-nuclear compartments in the U-2 OS *hyper*LOPIT dataset, which were validated by Human Protein Atlas annotations. An additional endosome-enriched phenotype was uncovered and Novelty TAGM robustly identified an overlapping phenotype in U-2 OS LOPIT-DC data, providing strong evidence for endosomal resolution. Further biologically relevant annotations were uncovered in these, as well as other datasets. For example, a group of vesicle-associated proteins involved in transport from the ER to the early Golgi was identified in the yeast *hyper*LOPIT dataset; resolution of the ribosomal subunits was identified in the fibroblast dataset, and separate nuclear, cytosolic and ribosomal annotations were identified in the DOM datasets.

A direct comparison with the state-of-the-art approach *phenoDisco* demonstrates clear differences between the approaches. Novelty TAGM, a fully Bayesian approach, quantifies uncertainty in both the number of newly discovered phenotypes and the individual protein-phenotype associations—*phenoDisco* provides no such information.

Improved annotation of the U-2 OS *hyper*LOPIT data allowed us to explore endosomal processes, which have not previously been considered with this dataset. We compare our results directly to immunofluorescence microscopy-based information from the HPA database and demonstrate the value of orthogonal spatial proteomics approaches to determine protein sub-cellular localisation. Our results provide insights on the sub-cellular localisation of proteins for which there is no information in the HPA Cell Atlas database.

During our analysis, we observed that the posterior similarity matrices have potential sub-clustering structures. Many known organelles and sub-cellular niches have sub-compartmentalisation, thus methodology to detect these sub-compartments is in preparation. Furthermore, we have observed that different experiments and different data modalities provide complementary results. Thus, integrative approaches to spatial proteomics analysis are also desired.

Our method is widely applicable within the field of spatial proteomics and builds upon state-of-the-art approaches. The computational algorithms presented here are disseminated as part of the Bioconductor project [85, 86] building on MS-based data structures provided in [87] and are available as part of the pRoloc suite, with all data provided in pRolocdata [88].

## Supporting information

S1 Text Analysis of further datasets, additional details of the statistical model, as well as a sensitivity analysis. (PDF)
