# Supplementary material for: A semi-supervised Bayesian approach for simultaneous protein sub-cellular localisation assignment and novelty detection
Source: PLoS Comput Biol. 2020 Nov 9;16(11):e1008288. doi: 10.1371/journal.pcbi.1008288 (PMC7707549; doi:10.1371/journal.pcbi.1008288)
Supplement: S1 Text — (PDF) [file pcbi.1008288.s001.pdf]

Supplement: A semi-supervised Bayesian approach for simultaneous protein sub-cellular localisation assignment and novelty detection

Oliver M. Crook * [1,2], Aikaterini Geladaki[1,3], Daniel J.H. Nightingale[1], Owen Vennard[1], Kathryn S. Lilley † [1], Laurent Gatto‡ [4], and Paul D.W. Kirk§ [2,5]

[1] *Cambridge Centre for Proteomics, Department of Biochemistry, University of Cambridge, Cambridge, UK*
[2] *MRC Biostatistics Unit, School of Clinical Medicine, University of Cambridge, Cambridge, UK*
[3] *Department of Genetics, Universtiy of Cambridge, Cambridge, UK*
[4] *de Duve Institute, UCLouvain, Avenue Hippocrate 75, 1200 Brussels, Belgium*
[5] *Cambridge Institute of Therapeutic Immunology & Infectious Disease (CITIID), Jeffrey Cheah Biomedical Centre, Cambridge Biomedical Campus, University of Cambridge, UK.*

October 27, 2020

Appendix

mESC chromatin enrichment validation

For the mESC dataset, Novelty TAGM reveals 8 new putative phenotypes. Novelty TAGM recovers the masked annotations with phenotype 2 having the enriched terms associated with chromatin, such as *chromatin* and *chromosome* ($p < 10^{-80}$). Phenotype 3 corresponds to a separate nuclear substructure with enrichment for the terms *nucleolus* ($p < 10^{-60}$) and *nuclear body* ($p < 10^{-30}$). Thus, in the mESC dataset Novelty TAGM confirms the chromatin enrichment preparation designed to separate chromatin and non-chromatin associated nuclear proteins [52]. In addition, phenotype 4 demonstrates enrichment for the ribosome annotation ($p < 10^{-35}$). Phenotype 1 is enriched for *centrosome* and *microtubule* annotations ($p < 10^{-15}$), though observing the PSM in Fig1 we can see there is much uncertainty in this phenotype. This uncertainty quantification can then be used as a basis for justifying additional expert annotation.

*omc25@cam.ac.uk
†ksl23@cam.ac.uk
‡laurent.gatto@uclouvain.be
§paul.kirk@mrc-bsu.cam.ac.uk

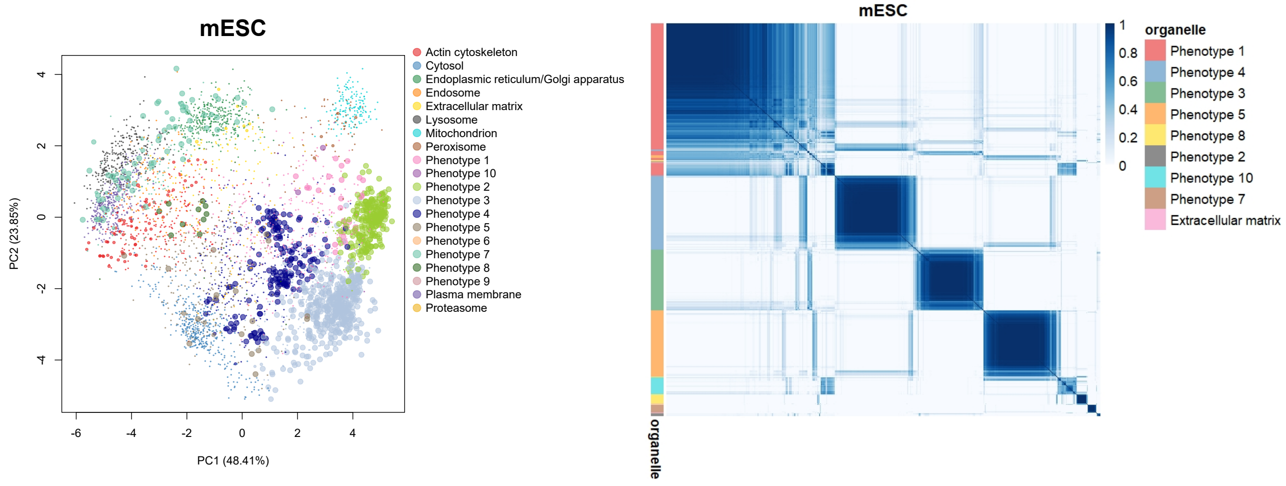

(a) (b)

Figure 1: (a) PCA plot of the *hyper*LOPIT mESC dataset. Points are scaled according to the discovery probability. (b) Heatmaps of the posterior similarity matrix derived from mESC data demonstrating the uncertainty in the clustering structure of the data. We have only plotted the proteins which have greater than 0.99 probability of belonging to a new phenotype and probability of being an outlier less than 0.95 for the mESC dataset to reduce the number of visualised proteins.

Uncovering additional annotations in fibroblast cells

HCMV-infected fibroblast cells

We apply Novelty TAGM to the dataset corresponding to the HCMV-infected fibroblast cells 24 hours post infection (hpi) [7], and discover 9 putative additional phenotypes (demonstrated in Fig2). Phenotype 2 contains a singleton protein and phenotypes $4, 6, 7, 8$ and 9 are not significantly enriched for any annotations. However, phenotype 3 is enriched for the *mitochondrial membrane* and *mitochondrial envelope* annotations ($p < 10^{-4}$); this is an addition to the already annotated mitochondrial class, indicating sub-mitochondrial resolution. Phenotype 1 is a mixed ribosomal/nuclear cluster with enrichment for *nucleoplasm* ($p < 10^{-5}$) and the *small ribosomal subunit* ($p < 10^{-4}$), which is distinct from phenotype 5 which is enriched for the *large ribosomal subunit* ($p < 10^{-10}$). This demonstrates unbiased separation of the two ribosomal subunits, which was overlooked in the original analysis [7].

Fibroblast cells without infection

Novelty TAGM reveals 7 putative phenotypes in the control fibroblast dataset [7]. Phenotypes $2, 4, 5, 6$ and 9 have no significantly enriched Gene Ontology terms (threshold $p = 0.01$). However, we observe that phenotype 3 is enriched with the *large ribosomal subunit* with significance at level $p < 10^{-7}$. Phenotype 1 represents a mixed *peroxisome* ($p < 10^{-2}$) and *mitochondrion* cluster ($p < 10^{-2}$), an unsurprising result since these organelles possess similar biochemical properties and therefore similar profiles during density gradient centrifugation-based fractionation [18, 29]. The differing number of confidently identified and biologically

relevant phenotypes discovered between the two fibroblast datasets could be down to the differing levels of structure between the two datasets. Indeed, it is evident from Fig2 that we see differing levels of clustering structure in these datasets.

Additional organellar map datasets

Mouse primary neurons

The mouse primary neuron dataset reveals 10 phenotypes after we apply Novelty TAGM. However, 8 of these phenotypes have no enriched GO annotations. This is likely a manifestation of the dispersed nature of this dataset, where the variability is generated by technical artefacts rather than biological signal. Despite this, Novelty TAGM is able to detect two relevant phenotypes: the first phenotype is enriched for *nucleolus* ($p < 0.01$); the second for *chromosome* ($p < 0.01$). This suggests additional annotations for this dataset.

HeLa cells (Hirst et. al 2018)

The HeLa dataset of [34], which we refer to as HeLa Hirst, reveals 7 phenotypes with at least 1 protein with discovery probability greater than 0.95. However, three of these phenotypes represent singleton proteins. Phenotype 1 reveals mixed cytosol/ribosomal annotations with the terms *cytosolic ribosome* ($p < 10^{-30}$) and *cytosolic part* ($p < 10^{-25}$) significantly over-represented. There are no further phenotypes with enriched annotations (threshold $p = 0.01$), except phenotype 2 which represents a mixed extracellular structure/cytosol cluster. For example, the terms *extracellular organelle* ($p < 10^{-13}$) and *cytosol* ($p < 10^{-10}$) are over-represented.

Handling label switching

Bayesian inference in mixture models suffers from an identifiability issue known as *label switching* - a phenomenon where the allocation labels can flip between runs of the algorithm [58, 66]. This occurs because of the symmetry of the likelihood function under permutations of these labels. We note that this only occurs in unsupervised or semi-supervised mixture models. This makes inference of the parameters in mixture models challenging. In our setting the labels for the known components do not switch, but for the new phenotypes label switching must occur. One standard approach to circumvent this issue is to form the so-called *posterior similarity matrix* (PSM) [22]. The PSM is an $N \times N$ matrix where the $(i, j)^{th}$ entry is the posterior probability that protein i and protein j reside in the same component. More precisely, if we let S denote the PSM and T denote the number of Monte-Carlo iterations then

$$S_{ij} = P(z_i = z_j | X, \boldsymbol{\theta}, \boldsymbol{\pi}, \kappa, \epsilon, \mathbf{M}, V) \approx \frac{1}{T} \sum_{t=1}^{T} \mathbb{I}(z_i^{(t)} = z_j^{(t)}), \tag{1}$$

where $\mathbb{I}$ denotes the indicator function. The PSM is clearly invariant to label switching and so avoids the issues arising from the *label switching* problem.

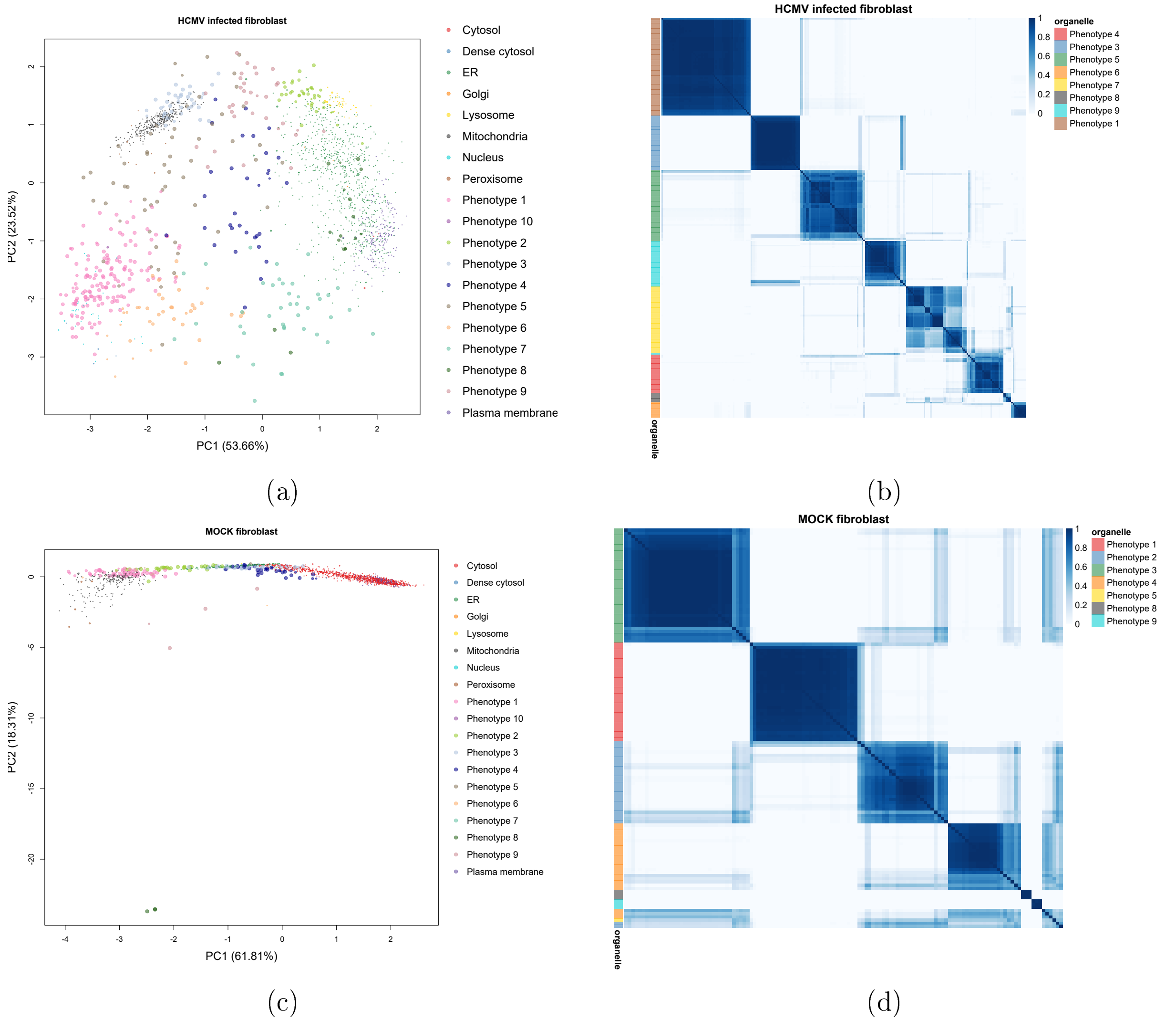

Figure 2: (a, c) PCA plots of the HCMV-infected fibroblast data 24 hpi and the mock fibroblast data 24 hpi. The points are coloured according to the organelle or proposed new phenotype and are scaled according to the discovery probability. (b, d) Heatmaps of the posterior similarity matrix derived from the infected fibroblast data and mock fibroblast data demonstrating the uncertainty in the clustering structure of the data. We have only plotted the proteins which have greater than 0.99 probability of belonging to a new phenotype and probability of being an outlier less than 0.95.

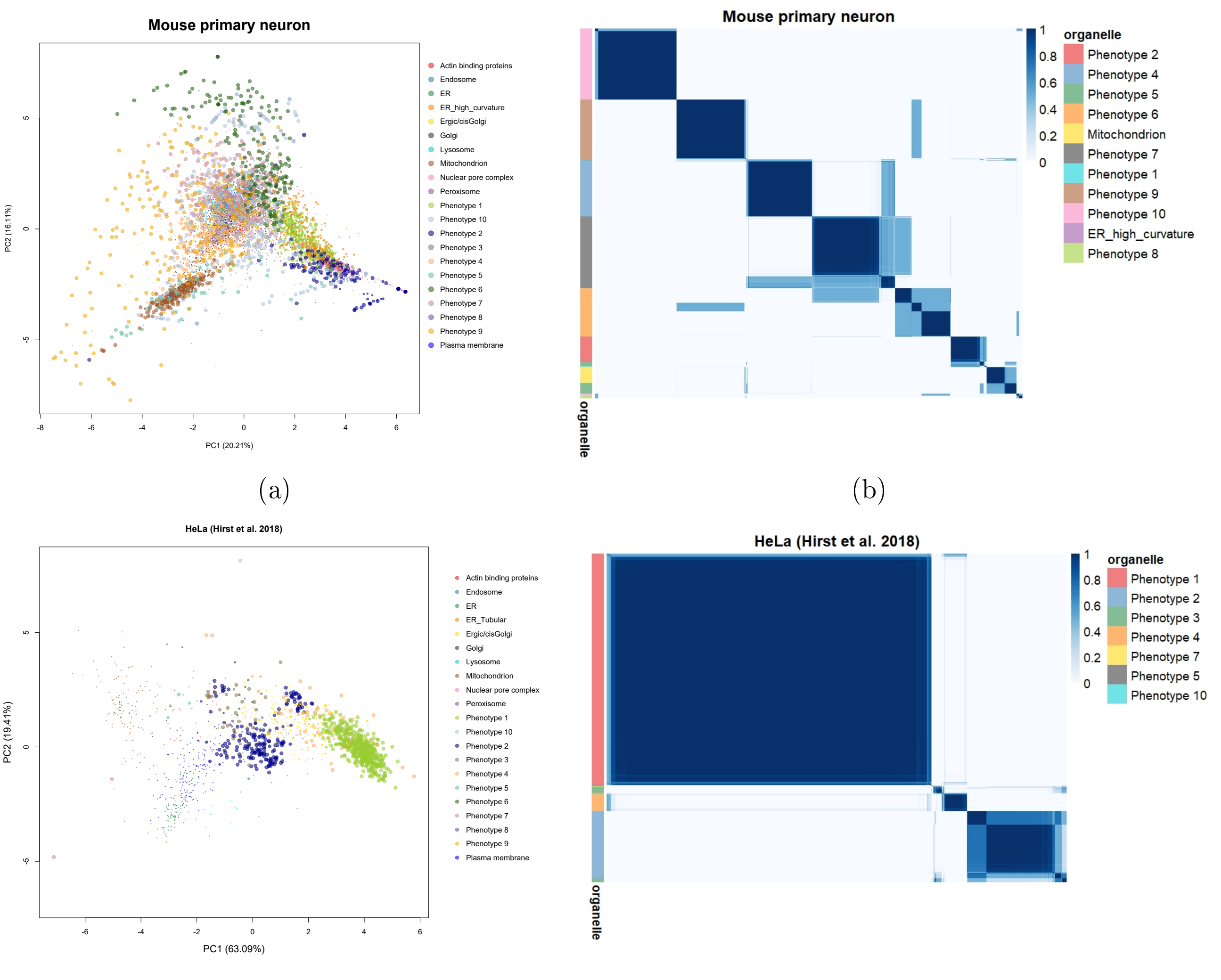

(a) (b)

(c) (d)

Figure 3: (a),(c) PCA plots of the mouse primary neuron data and HeLa Hirst data. The pointers are scaled according to their discovery probability. (b),(d) Heatmaps of the mouse neuron data and HeLa Hirst data. Only the proteins whose discovery probability is greater than 0.99 and outlier probability less than 0.95 (10^{-2} for the mouse primary neuron dataset to reduce the number of visualised proteins) are shown. The heatmaps demonstrate the uncertainty in the clustering structure present in the data.

Summarising posterior similarity matrices

To summarise the PSMs, we take the approach proposed by [22]. They proposed the adjusted Rand index (AR) [38, 56], a measure of cluster similarity, as a utility function and then we wish to find the allocation vector $\hat{z}$ that maximises the expected adjusted Rand index with respect to the true clustering z. Formally, we write

$$\hat{z} = \arg\max_{z^*} E[AR(z^*, z)|X, \boldsymbol{\theta}, \boldsymbol{\pi}, \kappa, \epsilon, \mathbf{M}, V], \tag{2}$$

which is known as the Posterior Expected Adjusted Rand index (PEAR). One obvious pitfall is that this quantity depends on the unknown true clustering z. However, this can be approximated from the MCMC samples:

$$PEAR \approx \frac{1}{T} \sum_{t=1}^{T} AR(z^*, z^{(t)}). \tag{3}$$

The space of all possible clustering over which to maximise is infeasibly large to explore. Thus we take an approach taken in [22] to propose candidate clusterings over which to maximise. Using hierarchical clustering with distance $1 - S_{ij}$, the PEAR criterion is computed for clusterings at every level of the hierarchy. The optimal clustering $\hat{z}$ is the allocation vector which maximises the PEAR.

Details of MCMC

The MCMC algorithm used in [14] is insufficient to handle inference of unknown phenotypes. As in [14], a collapsed Gibbs sampler approach is used, but a number of modifications are made. Firstly, to accelerate convergence of the algorithm half the proteins are initial allocated randomly amongst the new phenotypes. Secondly, the parameters for the new phenotypes are proposed from the prior. Throughout the same default prior choices are used as in [14].

Further details of endosomal proteins

For completeness, this appendix provides additional details and important literature on the proteins discussed in the main text.

First, P20339 (Rab5a) and P61020 (Rab5b) are two of the three isoforms of Rab5, a small GTPase which belongs to the Ras protein superfamily and is considered a master organiser of the endocytic system. Rab5a and Rab5b share a high level of amino acid sequence identity (approximately 85%) and are ubiquitously expressed in the mouse and human. Independently, these isoforms act as key regulators of clathrin-mediated endocytosis and early endosome dynamics by controlling the following processes *in vivo* and *in vitro*: (a) clathrin-coated vesicle formation at the cell surface; (b) endocytosed vesicle transport from the plasma membrane towards, and fusion with, early endosomes; (c) early endosome biogenesis and maintenance; (d) molecular motor-driven, microtubule-dependent early endosome motility along the endocytic route; (e) early endosome docking/tethering and homotypic fusion, and (f) Rab conversion and early-to-late endosome maturation [13, 28, 43, 59, 64, 82].

Rab5a and Rab5b play crucial roles in the internalisation and recycling/degradation of cell surface receptors such as EGFR (epidermal growth factor receptor), TfR (transferrin receptor) and several GPCRs (G-protein-coupled receptors) and integrins as well as peripheral plasma membrane-associated signalling molecules, thereby regulating important intracellular signal transduction pathways [5, 12, 45, 73]. We observe a mixed steady-state potential localisation between the endosome and PM for both Rab5a and Rab5b (Fig **??**D). According to previously published information, both Rab5a and Rab5b are mainly localised to (and considered well-established constituents of) the early endosome compartment but have also been detected on the PM and clathrin-coated vesicles, in support of our results [51, 64, 77]. Moreover, according to the HPA Cell Atlas, Rab5b resides in the vesicles (which, in this context, include the endosomes, lysosomes, peroxisomes and lipid droplets). There is no information regarding the sub-cellular location of Rab5a in this database.

Second, Q92738 (RN-tre) is a GTPase-activating protein (GAP) which controls the activity of several Rab GTPases. RN-tre is a major Rab5 (see above) regulator and therefore a key player in the organisation and dynamics of the endocytic pathway [28, 42]. This protein modulates the internalisation of and signal transduction mediated by cell surface receptors such as EGFR, TfR and β1 integrins [17, 42, 48, 55]. It also controls early endosome-to-Golgi retrograde transport and Golgi membrane organisation [32]. We observe a steady-state snapshot of the sub-cellular distribution of RN-tre with potential localisation to the endosome and PM (Fig **??**D). In line with these results, RN-tre has been shown to reside in Rab5-positive early endosomes at steady state, but has also been detected at the PM and focal adhesions [17, 28, 42, 48, 55]. There is no information concerning the sub-cellular localisation of RN-tre in the HPA Cell Atlas database.

Third, Q96L93 (KIF16B) is a plus end-directed molecular motor which belongs to the kinesin-3 protein family. This kinesin regulates early endosome motility along microtubules and is required for the establishment of the steady-state sub-cellular distribution of early endosomes as well as the balance between PM recycling and lysosome degradation of signal transducing cell surface receptors including EGFR and TfR [9, 35]. In neuronal cells, KIF16B plays an important role in the establishment of somatodendritic early endosome localisation and in the trafficking of AMPA and NGF receptors [21]. In epithelial cells, this protein controls the transcytosis of TfR from juxtanuclear recycling endosomes to apical recycling endosomes [6]. KIF16B is also involved in tubular endosome biogenesis and fission by regulating early endosome fusion [65]. Lastly, this kinesin has been shown to mediate biosynthetic Golgi-to-endosome transport of FGFR (fibroblast growth factor receptor)-carrying vesicles and thereby control FGFR cell surface presentation and signalling during in vivo mouse embryogenesis [74]. Our results indicate a mixed localisation to the endosome and PM for KIF16B (Fig **??**D). In line with our observations, it has been reported that this protein is associated with early endosome membranes at steady state in mouse and human cells [21, 35]. Additionally, it has been demonstrated that KIF16B co-localises with, and its spatial distribution and activity is regulated by, the small GTPase Rab5, whose isoforms Rab5a and Rab5b we also identified as potentially localised to the endosome and PM in the U-2 OS *hyper*LOPIT dataset (see above), on early endosomes [35, 65]. Taking the above into account, a mixed distribution between the endosome and PM is reflective of the molecular function of KIF16B. However, the HPA Cell Atlas database classifies KIF16B as a component of the mitochondria (Fig **??**B), contradicting our findings as well as previously published information

regarding the sub-cellular localisation and biological role of this protein. We speculate that this disagreement arises from the uncertainty associated with the specificity of the chosen antibody [71]. Indeed, the reliability of the mitochondrial annotation for KIF16B is classified as "uncertain" in this database.

Fourth, Q8NHG8 (ZNRF2) is an E3 ubiquitin ligase which has been shown to regulate mTOR signalling as well as lysosomal acidity and homeostasis in mouse and human cells [37]. This protein has been found to control the sub-cellular localisation and biological function of mTORC1, the V-ATPase and the Na+/K+-ATPase $\alpha 1$ [36, 37]. ZNRF2 is membrane-associated but can be released into the cytosol upon phosphorylation by various kinases [37]. We observe a mixed steady-state distribution between the endosome and PM for this protein (Fig ??D). In support of this result, we find that ZNRF2 has been detected on the endosomes, lysosomes, Golgi apparatus and PM according to the literature [1, 37]. There is no information in regard to the sub-cellular location of ZNRF2 in the HPA Cell Atlas database.

Fifth, O15498 (Ykt6) is a SNARE (soluble N-ethylmaleimide-sensitive factor attachment protein receptor) protein which is conserved from yeast to humans. This protein regulates a wide variety of intracellular trafficking and membrane tethering and fusion processes including ER-to-Golgi vesicular transport, intra-Golgi traffic, retrograde Golgi-to-ER transport, retrograde endosome-to-TGN (trans-Golgi network) trafficking, homotypic fusion of ER membranes, Golgi-to-PM transport and exosome/secretory vesicle-PM fusion, Golgi-to-vacuole traffic (in yeast), homotypic vacuole fusion (in yeast), autophagosome formation and autophagosome-lysosome fusion [20, 44, 49, 68, 69, 80]. Ykt6 lacks a transmembrane domain and is able to cycle between intracellular membranes and the cytosol in a palmitoylation- and farnesylation-dependent manner [25, 50]. The membrane-associated form of Ykt6 has been detected on the PM, ER, Golgi apparatus, endosomes, lysosomes, vacuoles (in yeast), and autophagosomes as part of various SNARE complexes [20, 25, 44, 49, 50, 68, 69, 80]. In line with this information, our results show a mixed sub-cellular distribution for Ykt6 with potential localisation to the endosome and cytosol (Fig ??D). The cytosolic localisation for Ykt6 is also supported by the HPA Cell Atlas annotation corresponding to this protein (Fig ??B), further reinforcing our findings.

Sixth, Q9NZN3 (EHD3) is another important regulator of endocytic trafficking and recycling. This protein promotes the biogenesis and stabilisation of tubular recycling endosomes by inducing early endosome membrane bending and tubulation [3, 33]. Additionally, EHD3 is essential for early endosome-to-recycling endosome transport, retrograde early endosome-to-Golgi traffic, Golgi apparatus morphology maintenance, and recycling endosome-to-PM transport [8, 31, 33, 53, 54]. It plays an important role in the recycling of cell surface receptors and the biosynthetic transport of lysosome proteins [8, 31, 53, 54]. We observe a mixed steady-state potential localisation to the endosome and PM for EHD3 (Fig ??D). Our results are in agreement with previously published studies which have reported that EHD3 is resident in the early endosomes and recycling endosomes at steady state [8, 31, 53, 54], and our PM localisation-related observation is supported by the HPA Cell Atlas-derived annotation for this protein (Fig ??B).

Our findings provide insights on the dynamic sub-cellular distribution of proteins which play important roles in development, physiology and disease. For example, Rab5/Rab5a has been identified as a master regulator of cancer cell migration, tumour invasion and

dissemination programs *in vitro* and *in vivo*. It has been demonstrated that Rab5/Rab5a expression is dysregulated in many invasive human cancers, Rab5/Rab5a is overexpressed in metastatic foci compared to the matched primary tumours, and Rab5/Rab5a activity critically promotes the acquisition of invasive properties by poorly invasive tumour cell types [19, 23, 45, 46, 51, 62, 72]. Several publications have reported that elevated Rab5/Rab5a expression correlates with, and is predictive of, increased local invasiveness and metastatic potential, as well as poor patient prognosis in a variety of human cancer types [19, 23, 26, 39, 51, 79, 81, 84]. Due to its established role in cancer progression and metastasis, Rab5/Rab5a is considered a fundamental cancer-associated protein and a potential diagnostic marker or therapeutic target [23, 39]. Recently, Rab5 was identified as a promising therapeutic target for colorectal cancer, as inhibition of Rab5 (and Rab7) activity led to elimination of colorectal cancer stem cells and disruption of colorectal cancer foci [70]. Moreover, individual ablation of Rab5a but also Rab5b was shown to impair the invasion and dissemination ability of different cancer cell types [23]. In addition to its important role in cancer, there is some evidence suggesting that Rab5a might also be involved in the early pathogenesis of Alzheimer's disease [10, 11, 60]. Lastly, the Rab5 machinery has also been identified as an important factor in several bacterial, parasitic and viral infections. Bacterial pathogens such as *Mycobacterium tuberculosis*, *Listeria monocytogenes*, *Tropheryma whipplei* and *Salmonella typhimurium* [47], as well as parasites such as *Leishmania donovani* have evolved specific subversion mechanisms with which they are able to control the intracellular distribution and/or activity of Rab5 and its effectors as a way to avoid neutralisation by the immune system or facilitate invasion [76]. *L. donovani* specifically controls the expression and function of the Rab5a isoform in this context [76]. Additionally, Rab5 was shown to participate in adenovirus endocytosis [57], both Rab5a and Rab5b were found to play functional roles in web formation and viral genome replication during HCV (hepatitis C virus) infection [67], and Rab5a was identified as a crucial target of HBV (hepatitis B virus) during HBV-related hepatocellular carcinoma pathogenesis [63].

Apart from Rab5a and Rab5b, the other proteins also possess demonstrated roles in development and disease. RN-tre is overexpressed in a subset of aggressive basal-like breast cancers, where high levels of this protein prevent the endocytosis and recycling of EGFR, leading to Akt overstimulation. In turn, Akt activity stabilises the glucose transporter GLUT1 at the cell membrane, resulting in an increase in glycolysis and cancer cell proliferation. RN-tre has been proposed as a potential therapeutic target for these types of breast cancer [2]. This protein also plays a functional role in infection, as it was shown to regulate the uptake and intracellular trafficking of Shiga toxins [24]. Furthermore, it has been reported that KIF16B is essential for early post-implantation mouse embryo development, as Kif16b-knockout animals display peri-implantation embryonic lethality [74]. In addition, recent studies have shown that ZNRF2 is overexpressed in human non-small cell lung cancer, osteosarcoma and papillary thyroid cancer, and that high levels of this protein are correlated with disease progression and poor patient prognosis in these cases [15, 78, 83]. Moreover, Ykt6 was found to be necessary for glycosome biogenesis and function in the kinetoplastid parasite *Trypanosoma brucei*, which causes African sleeping sickness, with Ykt6 ablation significantly reducing the viability of the parasite in both its pro-cyclic and bloodstream forms [4]. Finally, EHD3 has been identified as an essential factor for heart physiology [16].

Summary of convergence diagnostics

We provide a summary of convergence diagnostics using parallel chains analysis [30]. We compute the number of proteins allocated to the outlier component at each iteration of the Markov-chain and monitor this quantity for convergence. The $\hat{R}$ statistic between parallel chains in then computed and reported in the table below. A value of $\hat{R} < 1.2$ indicates convergence.

Convergence diagnostics for MCMC			
Dataset	Protocol	$\hat{R}$	Upper confidence Interval $\hat{R}$
mESC	*hyper*LOPIT	1.03	1.15
U-2 OS	*hyper*LOPIT	1.00	1.00
U-2 OS	LOPIT-DC	1.02	1.06
S. cerevisiae	*hyper*LOPIT	1.00	1.01
HCMV-infected fibroblast	Spatio-Temporal Proteomics	1.01	1.02
HCMV mock fibroblast	Spatio-Temporal Proteomics	1.03	1.08
HeLa [40]	DOM	1.07	1.21
Mouse primary neurons	DOM	1.04	1.13
HeLa [34]	DOM	1.02	1.06
HEK-293	LOPIT	1.00	1.01

Table 1: A table reporting convergence diagnostics for MCMC analysis

Prior specification and sensitivity

To complete the Bayesian specification, here we provide details of the priors on the model parameters. In the multivariate Gaussian components of the Novelty TAGM model, as with TAGM, a common and practical choice is the use of a normal-inverse-Wishart prior. That is,

$$\begin{aligned} \mu|\Sigma &\sim \mathcal{N}(\mu_0, \Sigma/\lambda_0) \\ \Sigma &\sim \mathcal{IW}(\nu_0, S_0) \\ &\propto |\Sigma|^{\frac{\nu_0+d+1}{2}} \exp\left[-\frac{1}{2}\text{trace}(\Sigma^{-1}S_0^{-1})\right], \end{aligned} \tag{4}$$

for each mixture component and where d is the dimension of the data. To complete this discussion, we need to specify the hyperparameters, μ_0, λ_0, ν_0 and S_0. We use diffusive priors that make minimal assumptions about the data, but they are set semi-empirically as to

obtain the correct scale of the data. The hyperparameters are selected as follows

$$\begin{aligned}
\mu_0 &= \frac{1}{n}\sum_{i=1}^{n} \mathbf{x_i}, \\
\lambda_0 &= 0.01, \\
\nu_0 &= d+2, \\
S_0 &= \frac{\text{diag }(\text{var}(X))}{K^{1/d}}.
\end{aligned} \tag{5}$$

The hyperparameters are interpreted in the following ways. The prior mean, μ_0, is the mean of the data. Then λ_0 is viewed as the number of observations with data μ_0 which are added to each component specific mean. This value is small to avoid strong prior influence. The marginal prior distribution (or prior predictive) for a cluster specific mean μ is given by a student's t-distribution. This can be observed by recalling that the student's t-distribution arises by marginalisation of the covariance from a normal distribution. Now, to ensure this t-distribution has finite covariance we require that $\nu_0 > d+1$. Thus, the choice presented here is the smallest integer value of ν_0 that ensures a finite covariance matrix. Hence, we have a well defined t-distribution with heavy tails. The empirically chosen scale matrix S_0 is chosen to roughly partition the range of the data into K balls of equal size. Previous work has shown that these priors lead to good predictive performance [14]. For $\boldsymbol{\pi}$, we take a conjugate symmetric Dirichlet prior with parameter β, so that $\pi_1, \ldots, \pi_{K_{max}} \sim \text{Dirichlet}(\beta)$. Note that to apply the principle of overfitted mixtures, we have to choose $\max_j \beta_j < d/2$ [61], which is satisfied in all examples by setting $\beta_j = 1$ for every j. Empirically Van Havre *et al.* [75] have recommended smaller values of $\beta_j \approx n^{-1}$ to encourage stronger shrinkage.

Sensitivity to the choice of β_j

To explore the sensitivity of our inferences to the specification of β_j, we considered setting $\beta_j = 0.1, 0.01$, as well as $\beta_j \approx n^{-1}$ for the mESC example, which in this case $n^{-1} \approx 0.0002$. As before, we hid nucleus, chromatin and ribosome annotations and sought to use our model to rediscover them. As we now summarily describe, we found that our results can be sensitive to the choice of β_j and hence it should be set carefully. For example when $\beta_j = 0.1$, we were unable to detect a ribosomal phenotype. Furthermore, there was a joint nucleus and chromatin phenotype, phenotype 1, rather than two distinct phenotypes. *Chromosome* was enriched for this phenotype ($p < 10^{-100}$), as well as nucleolus ($p < 10^{-60}$). When $\beta_j = 0.01$ the results were somewhat improved with a phenotype 1 enriched for *chromosome* ($p < 10^{-100}$) but phenotype 3 was enriched for cytosolic ribosome ($p < 10^{-48}$) and nucleolus ($p < 10^{-50}$). Setting $\beta_j = 0.0002$ provided the expected results with 3 distinct phenotypes for *chromatin* (phenotype 1) ($p < 10^{-100}$), nucleolus (phenotype 4) ($p < 10^{-50}$), and cytosolic ribsome (phenotype 3) ($p < 10^{-59}$), successfully matching our test components. Hence, based on these results, we would recommend either $\beta_j = 1$ or $\beta_j \approx n^{-1}$ depending on the desired amount of shrinkage.

Impact of reducing the proportion of labelled proteins

In all the examples we considered previously, the proportion of labelled proteins is roughly 20% of the total number of proteins. To assess the impact of the relative proportion of labelled and unlabelled proteins, we reconsidered our mESC example, where the goal was to detect ribosomal, nuclear and chromatin niches without annotation. In addition to masking these annotations as test components, we also masked, uniformly at random, an additional $10\%, 20\%$ and 50% of labelled proteins and assessed our ability to rediscover the ribosomal, nuclear and chromatin testing classes.

Briefly, we were able to rediscover two distinct phenotypes according to two nuclear clusters in all cases. When we masked 10% of the labels, the enrichments for the two nuclear phenotypes were chromosome ($p < 10^{-99}$) and nucleolus ($p < 10^{-59}$), the results were the same when we removed 20% and 50% of labels. However, only in the scenario were 20% of the labels were hidden did we find a ribosome enriched phenotype ($p < 10^{-30}$). In the other cases, the ribosome clustered with the other large protein complex: the proteasome. This reflects the similar biochemical properties of these subcellular niches. Furthermore, removing annotations renders the proteasome profile less well defined, resulting in a more diffuse cluster. In practice, careful quality control would mitigate these scenarios [27]. In applications where there are very few annotated niches and the analysis is close to the unsupervised setting, it may be valuable to increase $K_{novelty}$ above 10 - others have found $n/2$ to work well [41].

References

[1] Araki, T. et al. (2003). Znrf proteins constitute a family of presynaptic e3 ubiquitin ligases. *Journal of Neuroscience*, **23**(28), 9385–9394.

[2] Avanzato, D. et al. (2018). High usp6nl levels in breast cancer sustain chronic akt phosphorylation and glut1 stability fueling aerobic glycolysis. *Cancer research*, **78**(13), 3432–3444.

[3] Bahl, K. et al. (2016). Ehd3 protein is required for tubular recycling endosome stabilization, and an asparagine-glutamic acid residue pair within its eps15 homology (eh) domain dictates its selective binding to npf peptides. *Journal of Biological Chemistry*, **291**(26), 13465–13478.

[4] Banerjee, H. et al. (2017). Involvement of snare protein ykt6 in glycosome biogenesis in trypanosoma brucei. *Molecular and biochemical parasitology*, **218**, 28–37.

[5] Bastin, G. et al. (2013). Rab family proteins regulate the endosomal trafficking and function of rgs4. *Journal of Biological Chemistry*, **288**(30), 21836–21849.

[6] Bay, A. E. P. et al. (2013). The kinesin kif16b mediates apical transcytosis of transferrin receptor in ap-1b-deficient epithelia. *The EMBO journal*, **32**(15), 2125–2139.

[7] Beltran, P. M. J. et al. (2016). A portrait of the human organelle proteome in space and time during cytomegalovirus infection. *Cell systems*, **3**(4), 361–373.

[8] Cabasso, O. et al. (2015). Sumoylation of ehd3 modulates tubulation of the endocytic recycling compartment. *PloS one*, **10**(7), e0134053.

[9] Carlucci, A. et al. (2010). Ptpd1 supports receptor stability and mitogenic signaling in bladder cancer cells. *Journal of biological chemistry*, **285**(50), 39260–39270.

[10] Cataldo, A. M. et al. (1997). Increased neuronal endocytosis and protease delivery to early endosomes in sporadic alzheimer's disease: neuropathologic evidence for a mechanism of increased β-amyloidogenesis. *Journal of Neuroscience*, **17**(16), 6142–6151.

[11] Cataldo, A. M. et al. (2000). Endocytic pathway abnormalities precede amyloid β deposition in sporadic alzheimer's disease and down syndrome: differential effects of apoe genotype and presenilin mutations. *The American journal of pathology*, **157**(1), 277–286.

[12] Chen, P.-I. et al. (2009). Rab5 isoforms differentially regulate the trafficking and degradation of epidermal growth factor receptors. *Journal of Biological Chemistry*, **284**(44), 30328–30338.

[13] Chen, P.-I. et al. (2014). Rab5 isoforms orchestrate a "division of labor" in the endocytic network; rab5c modulates rac-mediated cell motility. *PloS one*, **9**(2), e90384.

[14] Crook, O. M. et al. (2018). A bayesian mixture modelling approach for spatial proteomics. *PLOS Computational Biology*, **14**(11), 1–29.

[15] Cui, Z. et al. (2019). Long non-coding rna ttn-as1 facilitates tumorigenesis of papillary thyroid cancer through modulating mir-153-3p/znrf2 axis. *The journal of gene medicine*, page e3083.

[16] Curran, J. et al. (2014). Ehd3-dependent endosome pathway regulates cardiac membrane excitability and physiology. *Circulation research*, **115**(1), 68–78.

[17] De Franceschi, N. et al. (2015). Integrin traffic–the update. *J Cell Sci*, **128**(5), 839–852.

[18] Dealtry, G. B. et al. (1992). *Cell biology labfax*. Distributed in the United States and Canada by Academic Press.

[19] Díaz, J. et al. (2014). Rab5 is required in metastatic cancer cells for caveolin-1-enhanced rac1 activation, migration and invasion. *J Cell Sci*, **127**(11), 2401–2406.

[20] Dilcher, M. et al. (2001). Genetic interactions with the yeast q-snare vti1reveal novel functions for the r-snare ykt6. *Journal of Biological Chemistry*, **276**(37), 34537–34544.

[21] Farkhondeh, A. et al. (2015). Characterizing kif16b in neurons reveals a novel intramolecular "stalk inhibition" mechanism that regulates its capacity to potentiate the selective somatodendritic localization of early endosomes. *Journal of Neuroscience*, **35**(12), 5067–5086.

[22] Fritsch, A. et al. (2009). Improved criteria for clustering based on the posterior similarity matrix. *Bayesian Anal.*, **4**(2), 367–391.

[23] Frittoli, E. et al. (2014). A rab5/rab4 recycling circuitry induces a proteolytic invasive program and promotes tumor dissemination. *J Cell Biol*, **206**(2), 307–328.

[24] Fuchs, E. et al. (2007). Specific rab gtpase-activating proteins define the shiga toxin and epidermal growth factor uptake pathways. *The Journal of cell biology*, **177**(6), 1133–1143.

[25] Fukasawa, M. et al. (2004). Localization and activity of the snare ykt6 determined by its regulatory domain and palmitoylation. *Proceedings of the National Academy of Sciences*, **101**(14), 4815–4820.

[26] Fukui, K. et al. (2007). Expression of rab5a in hepatocellular carcinoma: possible involvement in epidermal growth factor signaling. *Hepatology Research*, **37**(11), 957–965.

[27] Gatto, L. et al. (2019). Assessing sub-cellular resolution in spatial proteomics experiments. *Current opinion in chemical biology*, **48**, 123–149.

[28] Gautreau, A. et al. (2014). Function and regulation of the endosomal fusion and fission machineries. *Cold Spring Harbor perspectives in biology*, **6**(3), a016832.

[29] Geladaki, A. et al. (2019). Combining lopit with differential ultracentrifugation for high-resolution spatial proteomics. *Nature Communications*, **10**, 331.

[30] Gelman, A. et al. (1992). Inference from iterative simulation using multiple sequences. *Statistical science*, pages 457–472.

[31] George, M. et al. (2007). Shared as well as distinct roles of ehd proteins revealed by biochemical and functional comparisons in mammalian cells and c. elegans. *BMC cell biology*, **8**(1), 3.

[32] Haas, A. K. et al. (2007). Analysis of gtpase-activating proteins: Rab1 and rab43 are key rabs required to maintain a functional golgi complex in human cells. *Journal of cell science*, **120**(17), 2997–3010.

[33] Henmi, Y. et al. (2016). Phosphatidic acid induces ehd3-containing membrane tubulation and is required for receptor recycling. *Experimental cell research*, **342**(1), 1–10.

[34] Hirst, J. et al. (2018). Role of the ap-5 adaptor protein complex in late endosome-to-golgi retrieval. *PLoS biology*, **16**(1), e2004411.

[35] Hoepfner, S. et al. (2005). Modulation of receptor recycling and degradation by the endosomal kinesin kif16b. *Cell*, **121**(3), 437–450.

[36] Hoxhaj, G. et al. (2012). Znrf2 is released from membranes by growth factors and, together with znrf1, regulates the na+/k+ atpase. *J Cell Sci*, **125**(19), 4662–4675.

[37] Hoxhaj, G. et al. (2016). The e3 ubiquitin ligase znrf2 is a substrate of mtorc1 and regulates its activation by amino acids. *elife*, **5**, e12278.

[38] Hubert, L. et al. (1985). Comparing partitions. *Journal of Classification*, **2**(1), 193–218.

[39] Igarashi, T. et al. (2017). Association of rab5 overexpression in pancreatic cancer with cancer progression and poor prognosis via e-cadherin suppression. *Oncotarget*, **8**(7), 12290.

[40] Itzhak, D. N. et al. (2016). Global, quantitative and dynamic mapping of protein subcellular localization. *Elife*, **5**, e16950.

[41] Kirk, P. et al. (2012). Bayesian correlated clustering to integrate multiple datasets. *Bioinformatics*, **28**(24), 3290–3297.

[42] Lanzetti, L. et al. (2000). The eps8 protein coordinates egf receptor signalling through rac and trafficking through rab5. *Nature*, **408**(6810), 374.

[43] Law, F. et al. (2017). The vps34 pi3k negatively regulates rab-5 during endosome maturation. *J Cell Sci*, **130**(12), 2007–2017.

[44] Linnemannstöns, K. et al. (2018). Ykt6 membrane-to-cytosol cycling regulates exosomal wnt secretion. *bioRxiv*, page 485565.

[45] Liu, B. et al. (2015). Cmtm7 knockdown increases tumorigenicity of human non-small cell lung cancer cells and egfr-akt signaling by reducing rab5 activation. *Oncotarget*, **6**(38), 41092.

[46] Liu, S.-s. et al. (2011). Knockdown of rab5a expression decreases cancer cell motility and invasion through integrin-mediated signaling pathway. *Journal of biomedical science*, **18**(1), 58.

[47] Madan, R. et al. (2008). Sope-mediated recruitment of host rab5 on phagosomes inhibits salmonella transport to lysosomes. In *Autophagosome and Phagosome*, pages 417–437. Springer.

[48] Martinu, L. et al. (2002). Endocytosis of epidermal growth factor receptor regulated by grb2-mediated recruitment of the rab5 gtpase-activating protein rn-tre. *Journal of Biological Chemistry*, **277**(52), 50996–51002.

[49] Matsui, T. et al. (2018). Autophagosomal ykt6 is required for fusion with lysosomes independently of syntaxin 17. *J Cell Biol*, **217**(8), 2633–2645.

[50] Meiringer, C. T. et al. (2008). Depalmitoylation of ykt6 prevents its entry into the multivesicular body pathway. *Traffic*, **9**(9), 1510–1521.

[51] Mendoza, P. et al. (2013). Rab5 activation promotes focal adhesion disassembly, migration and invasiveness in tumor cells. *J Cell Sci*, **126**(17), 3835–3847.

[52] Mulvey, C. M. et al. (2017). Using hyperLOPIT to perform high-resolution mapping of the spatial proteome. *Nature Protocols*, **12**(6), 1110–1135.

[53] Naslavsky, N. et al. (2006). Interactions between ehd proteins and rab11-fip2: a role for ehd3 in early endosomal transport. *Molecular biology of the cell*, **17**(1), 163–177.

[54] Naslavsky, N. et al. (2009). Ehd3 regulates early-endosome-to-golgi transport and preserves golgi morphology. *Journal of cell science*, **122**(3), 389–400.

[55] Palamidessi, A. et al. (2013). The gtpase-activating protein rn-tre controls focal adhesion turnover and cell migration. *Current biology*, **23**(23), 2355–2364.

[56] Rand, W. M. (1971). Objective criteria for the evaluation of clustering methods. *Journal of the American Statistical association*, **66**(336), 846–850.

[57] Rauma, T. et al. (1999). rab5 gtpase regulates adenovirus endocytosis. *Journal of virology*, **73**(11), 9664–9668.

[58] Richardson, S. et al. (1997). On bayesian analysis of mixtures with an unknown number of components (with discussion). *Journal of the Royal Statistical Society: series B (statistical methodology)*, **59**(4), 731–792.

[59] Rink, J. et al. (2005). Rab conversion as a mechanism of progression from early to late endosomes. *Cell*, **122**(5), 735–749.

[60] Rosenfeld, J. L. et al. (2001). Lysosome proteins are redistributed during expression of a gtp-hydrolysis-defective rab5a. *Journal of cell science*, **114**(24), 4499–4508.

[61] Rousseau, J. et al. (2011). Asymptotic behaviour of the posterior distribution in overfitted mixture models. *Journal of the Royal Statistical Society: Series B (Statistical Methodology)*, **73**(5), 689–710.

[62] Saitoh, S. et al. (2017). Rab5-regulated endocytosis plays a crucial role in apical extrusion of transformed cells. *Proceedings of the National Academy of Sciences*, **114**(12), E2327–E2336.

[63] Sheng, Y. et al. (2014). Downregulation of mir-101-3p by hepatitis b virus promotes proliferation and migration of hepatocellular carcinoma cells by targeting rab5a. *Archives of virology*, **159**(9), 2397–2410.

[64] Simonsen, A. et al. (1998). Eea1 links pi (3) k function to rab5 regulation of endosome fusion. *Nature*, **394**(6692), 494.

[65] Skjeldal, F. M. et al. (2012). The fusion of early endosomes induces molecular-motor-driven tubule formation and fission. *J Cell Sci*, **125**(8), 1910–1919.

[66] Stephens, M. (2000). Dealing with label switching in mixture models. *Journal of the Royal Statistical Society: Series B (Statistical Methodology)*, **62**(4), 795–809.

[67] Stone, M. et al. (2007). Participation of rab5, an early endosome protein, in hepatitis c virus rna replication machinery. *Journal of virology*, **81**(9), 4551–4563.

[68] Tai, G. et al. (2004). Participation of the syntaxin 5/ykt6/gs28/gs15 snare complex in transport from the early/recycling endosome to the trans-golgi network. *Molecular biology of the cell*, **15**(9), 4011–4022.

[69] Takáts, S. et al. (2018). Non-canonical role of the snare protein ykt6 in autophagosome-lysosome fusion. *PLoS genetics*, **14**(4), e1007359.

[70] Takeda, M. et al. (2019). Disruption of endolysosomal rab5/7 efficiently eliminates colorectal cancer stem cells. *Cancer research*, pages canres–2192.

[71] Thul, P. J. et al. (2017). A subcellular map of the human proteome. *Science*, **356**(6340), eaal3321.

[72] Torres, V. A. et al. (2010). Rab5 mediates caspase-8–promoted cell motility and metastasis. *Molecular biology of the cell*, **21**(2), 369–376.

[73] Trischler, M. et al. (1999). Biochemical analysis of distinct rab5-and rab11-positive endosomes along the transferrin pathway. *J Cell Sci*, **112**(24), 4773–4783.

[74] Ueno, H. et al. (2011). Kif16b/rab14 molecular motor complex is critical for early embryonic development by transporting fgf receptor. *Developmental cell*, **20**(1), 60–71.

[75] Van Havre, Z. et al. (2015). Overfitting bayesian mixture models with an unknown number of components. *PloS one*, **10**(7), e0131739.

[76] Verma, J. K. et al. (2017). Leishmania donovani resides in modified early endosomes by upregulating rab5a expression via the downregulation of mir-494. *PLoS pathogens*, **13**(6), e1006459.

[77] Woodman, P. G. (2000). Biogenesis of the sorting endosome: the role of rab5. *Traffic*, **1**(9), 695–701.

[78] Xiao, Q. et al. (2017). Microrna-100 suppresses human osteosarcoma cell proliferation and chemo-resistance via znrf2. *Oncotarget*, **8**(21), 34678.

[79] Yang, P.-S. et al. (2011). Rab5a is associated with axillary lymph node metastasis in breast cancer patients. *Cancer science*, **102**(12), 2172–2178.

[80] Yong, C. Q. Y. et al. (2019). Another longin snare for autophagosome-lysosome fusion-how does ykt6 work? *Autophagy*, **15**(2), 352–357.

[81] Yu, L. et al. (1999). Differential expression of rab5a in human lung adenocarcinoma cells with different metastasis potential. *Clinical & experimental metastasis*, **17**(3), 213–219.

[82] Zerial, M. et al. (2001). Rab proteins as membrane organizers. *Nature reviews Molecular cell biology*, **2**(2), 107.

[83] Zhang, X. et al. (2016). The role of znrf2 in the growth of non-small cell lung cancer. *European review for medical and pharmacological sciences*, **20**, 4011–4017.

[84] Zhao, Z. et al. (2010). Rab5a overexpression promoting ovarian cancer cell proliferation may be associated with appl1-related epidermal growth factor signaling pathway. *Cancer science*, **101**(6), 1454–1462.
